# Early evidence of fire in south-western Europe: the Acheulean site of Gruta da Aroeira (Torres Novas, Portugal)

**DOI:** 10.1038/s41598-020-68839-w

**Published:** 2020-07-21

**Authors:** Montserrat Sanz, Joan Daura, Dan Cabanes, Natalia Égüez, Ángel Carrancho, Ernestina Badal, Pedro Souto, Filipa Rodrigues, João Zilhão

**Affiliations:** 1 Grup de Recerca del Quaternari (GRQ-SERP), Departament d’Història i Arqueologia, Universitat de Barcelona, Carrer Montalegre, 6, 08001 Barcelona, Spain; 2 UNIARQ-Centro de Arqueologia da Universidade de Lisboa, Faculdade de Letras, Universidade de Lisboa, 1600-214 Lisbon, Portugal; 3 Department of Anthropology, Rutgers University, Biological Sciences Building, 32 Bishop Street, New Brunswick, NJ 08901 USA; 4 Archaeological Micromorphology and Biomarkers, AMBI Lab, Instituto Universitario de Bio-Orgánica Antonio González, Universidad de La Laguna, 38206 Tenerife, Spain; 5 Área de Prehistoria. Dpto. Historia, Geografía y Comunicación. Edificio I+D+i, Universidad de Burgos, Plaza Misael Bañuelos S/N, 09001 Burgos, Spain; 6 PREMEDOC-GIUV2015-213, Departament de Prehistòria, Arqueologia i Història Antiga, Universitat de València, Av. Blasco Ibañez 28, 46010 Valencia, Spain; 7 STEA, Sociedade Torrejana de Espeleologia e Arqueologia, Quinta da Lezíria, 2350-510 Torres Novas, Portugal; 8 Departament d’Història i Arqueologia, Universitat de Barcelona, Carrer Montalegre, 6, 08001 Barcelona, Spain; 9 Institució Catalana de Recerca i Estudis Avançats (ICREA), Passeig Lluís Companys 23, 08010 Barcelona, Spain

**Keywords:** Archaeology, Archaeology

## Abstract

The site of Gruta da Aroeira (Torres Novas, Portugal), with evidence of human occupancy dating to ca. 400 ka (Marine Isotope Stage 11), is one of the very few Middle Pleistocene localities to have provided a fossil hominin cranium associated with Acheulean bifaces in a cave context. The multi-analytic study reported here of the by-products of burning recorded in layer X suggests the presence of anthropogenic fires at the site, among the oldest such evidence in south-western Europe. The burnt material consists of bone, charcoal and, possibly, quartzite cobbles. These finds were made in a small area of the cave and in two separate occupation horizons. Our results add to our still-limited knowledge about the controlled use of fire in the Lower Palaeolithic and contribute to ongoing debates on the behavioural complexity of the Acheulean of Europe.

## Introduction

Controlling the use of fire was a technological milestone in human evolution that broadened diet, expanded the ecological range, and provided a powerful defensive and offensive tool^1–4^. It required a complex mind, capable of predicting fire behaviour and fuel needs, and imposed high energetic costs^5^. Identifying the point in human evolution at which the benefits of fire outweighed its costs is one of Palaeoanthropology’s Big Issues.

Preserved hearths containing a combination of combustion residues, including ash, charred plant or animal remains, thermally altered sediments, and burnt artefacts provide direct evidence of the controlled use of fire^6^. However, the beginnings of pyrotechnology remain controversial because its remains are easily altered and their identification in the archaeological record can be hindered by taphonomic biases. In open-air sites, wind, rainfall or other erosion agents may erase the evidence for burning^4^, while wildfires can alter buried remnants and confound depositional and post-depositional events^7,8^. In caves, the probability that fire remains reflect in-situ events is higher and so the presence of by-products of burning can be considered good evidence that they result from human activity^9^. However, the preservation of fire remains in caves and rock shelters can also be affected by diagenetic processes related to the accumulation of bat guano and bird pellets^10,11^.

The earliest evidence suggestive of interaction between hominins and fire is found in Africa, ca. 1.5 Ma—e.g., the rubefied sediments found at Koobi Fora and Chesowanja (Kenya)^2,12,13^ and the burnt bones from Swartkrans (South Africa)^14^. At Wonderwerk (South Africa), ca. 1 Ma ago, thermally modified material (ashed plant remains and burnt bone, but no hearths) has been documented in association with Acheulean tools^15^.

In the Near East, the co-occurrence of charred plant remains and thermally altered lithics has been claimed to represent direct evidence of anthropogenic fires at the open-air site of Gesher Benot Ya’aqov (Israel, ca. 800 ka)^16,17^. Here, discrete concentrations of burnt flint micro-artefacts, taken as proxies for hearths, were also found^18^, but the earliest undisputed evidence dates to 420–200 ka, as exemplified by the in-situ fireplaces (wood ash) associated with burnt bones and lithics found in the upper deposits of Qesem Cave (Israel)^19,20^.

In Europe, claims for fire before MIS (Marine Isotope Stage) 11 rely on indirect evidence. This includes dispersed charcoal fragments in caves, including those found in level TE19 G at the Sima del Elefante site (Atapuerca, Spain)^21^, or in open-air sites, such as Boxgrove (UK)^22^. Thermally altered materials, such as bone and chert, have also been found in Cueva Negra del Rio Quípar in Spain^23,24^, among other sites^9^. However, the anthropogenic nature of these remains is controversial because it cannot be excluded that they are in secondary position and originate in wildfires outside the cave^25^.

**Figure 1:**
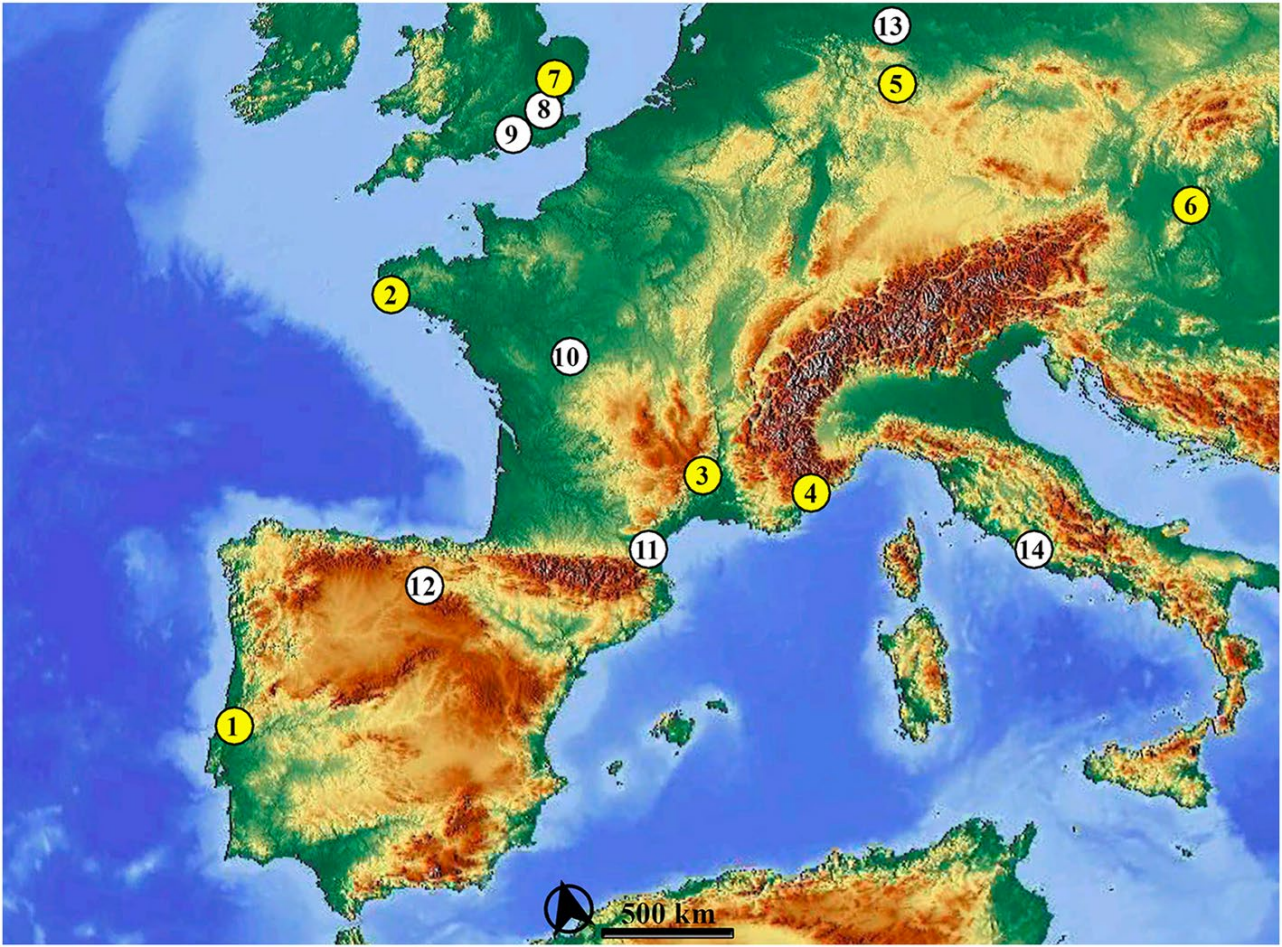
Map showing the location of the main Acheulean and MIS 11-MIS 9 sites in central and western Europe indicating the absence (white dots) or presence (yellow dots) of fire. 1: Gruta da Aroeira (Portugal). 2: Menez-Dregan (France). 3: Orgnac 3 (France). 4: Terra Amata (France). 5: Bilzingsleben (Germany). 6: Vérteszöllös (Hungary). 7: Beeches Pit (United Kingdom). 8. Swanscombe (United Kingdom). 9: Boxgrove (United Kingdom). 10: La Grande Vallée (France). 11. Caune de l’Arago (France). 12: Sierra de Atapuerca sites (Spain). 13: Schöningen (Germany). 14: Torre in Pietra (Italy). Map extracted from OpenStreetMap © licensed under ODdL 1.0 (https://www.openstreetmap.org/copyright) by the OpenStreetMap Foundation (OSMF). ©OpenStreetMap contributors.

Evidence for hearths or burning by-products dated to between MIS 11 and MIS 9 in Europe comes from open-air sites only. Examples are: burnt flint, charred bone and seemingly thermally altered sediments at Beeches Pit (UK)^26^; burnt material, charcoal and fire remnants at Terra Amata (France)^27^; burnt bones and possible hearths at Vérteszöllös (Hungary)^9^; burnt cherts at La Grande Vallée (France)^28^. Fire remnants in caves are scarce; the coastal site of Menez-Dregan 1 (France) yielded one of the few and amongst the oldest fireplaces (end of MIS 12 or beginning of MIS 11)^29,30^, and fireplaces have also been reported (but not described in detail) from Orgnac 3^9,31^ (Fig. 1).

It seems plausible that the control of fire spread across Europe synchronously with the Acheulean technology, ca. 500–600 ka, reaching Iberia ca. 450 ka^32,33^. Some authors associate the Acheulean expansion with an “out-of-Africa” dispersal occurring subsequent to a speciation event^34^, but the human fossils from the European Middle Pleistocene are considerably diverse^35^. Thus, while the Sima de los Huesos (Atapuerca) and other European hominins are claimed to belong in the Neandertal clade, the penecontemporaneous Caune de l’Arago fossils have been attributed to either *Homo heidelbergensis* or a subspecies of *Homo erectus*^36^.

The combination of traits displayed by the Gruta da Aroeira (Portugal) cranium Aroeira 3, recovered in association with Acheulean bifaces and dated to MIS 11, has brought additional complexity to this picture^36^. Ongoing research and excavations at this site—a Palaeolithic locality in the complex of archaeological sites associated with the karst spring of the Almonda river, in central Portugal (Supplementary Text S1 and Supplementary Fig. S1)—have also yielded evidence that we interpret as one of the earliest examples of anthropogenic fire recorded in SW Europe to date. Our interpretation, reported here, is based on the study of the by-products of burning (charcoal, bones, sediments and lithics) from the site’s layer X (mostly, from sub-layer Xc), employing soil micromorphology, organic chemistry, Fourier Transform Infrared spectroscopy (FTIR), magnetic properties, elemental composition, energy-dispersive X-ray spectroscopy, and the analysis of spatial distribution patterns. The samples that have been analysed are listed in Supplementary Table S1 and will henceforth be designated by their “Sample ID” reference.

## Results

### Faunal remains

#### Zooarchaeology and taphonomy

**Figure 2:**
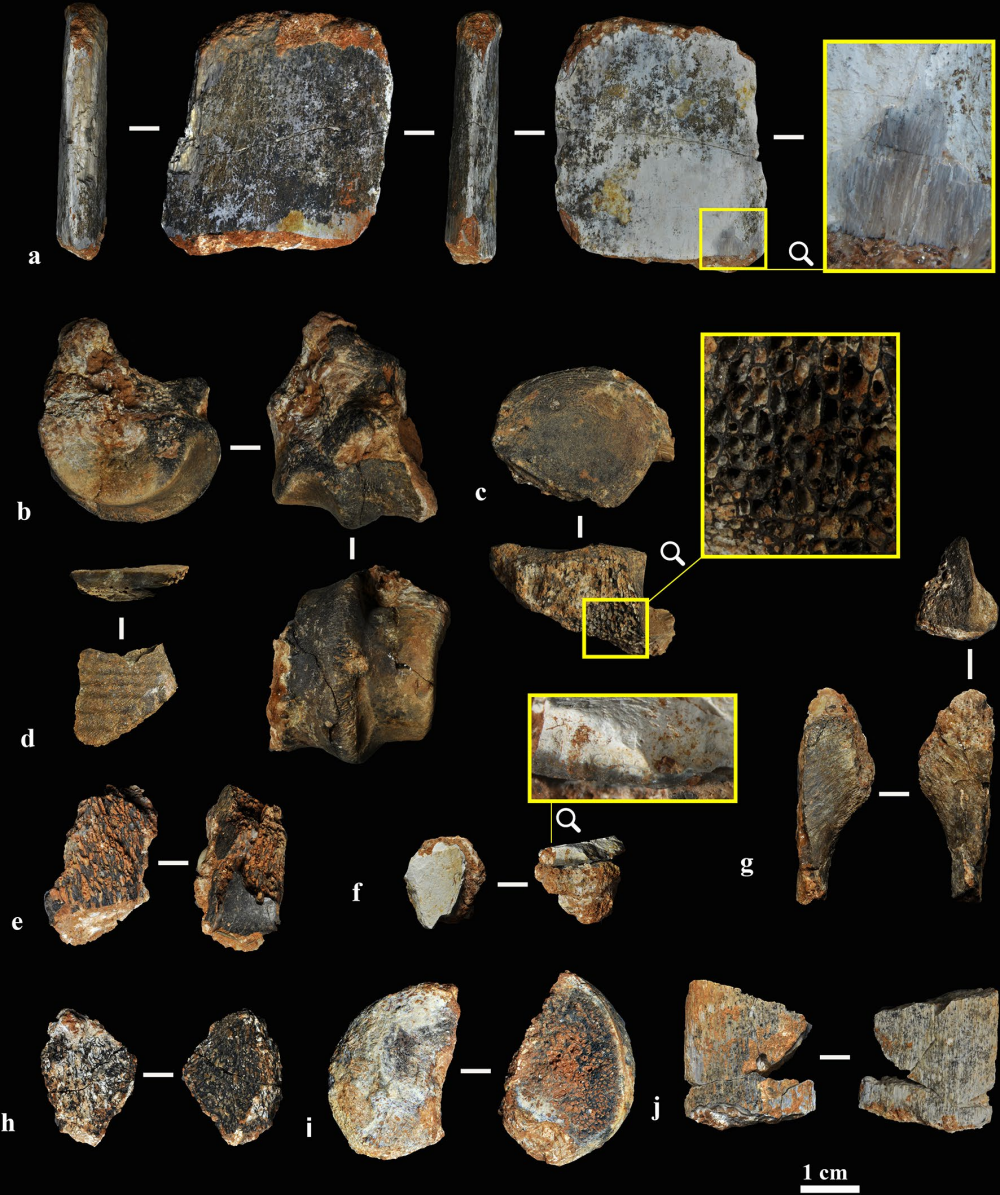
Burnt bones. (**a**) Fully calcined rib, blue-grey and white in colour (sample ID #13). (**b**) Distal condyle of a deer metapodial, fully carbonized (sample ID #3). (**c**) Fully carbonized vertebral body (sample ID #30). (**d**) Partially carbonized tortoise bone plate (sample ID #6). (**e**) Fully carbonized spongy fragment (sample ID #14). (**f**) Fully calcined flat bone (sample ID #15). (**g**) Fully carbonized flat bone (sample ID #2). (**h**) Fully carbonized spongy fragment (sample ID #20). (**i**) Fully carbonized fragmented epiphysis (sample ID #4). (**j**) Fully calcined rib (sample ID #27).

The six-grade colour scale proposed by Stiner^7^ was applied to the burnt bones from layer X (n = 43) (Supplementary Table S1). The different degrees of burning are indicative of the intensity of the alteration by heat (Supplementary Table S2 and Fig. 2): 22 fragments were fully carbonized (51%), seven were more than half carbonized (16%), six were more than half calcined (14%), four were fully calcined and completely white (9%), and two were slightly calcined (5%). Three fragments (sample ID #19, #27 and #13) also present manganese coatings with a weak degree of alteration. Dissolution is only observed in one (sample ID #13). No weathered bones have been recorded.

The burnt bones are highly fragmented, hindering assignment to species or genus (Fig. 2). The mean burnt bone length is ca. 20 mm with a maximum of 62 mm and a minimum of 7 mm. Two fragments can be ascribed to deer (a distal metapodial condyle and a thoracic vertebra), one to tortoise (a carapace fragment), and another to a small vertebrate (a mandible). No direct anthropogenic modification of these bones (e.g. cut-marks, diagnostic elements of bone breakage, etc.) could be identified.

#### FTIR on bones

**Figure 3:**
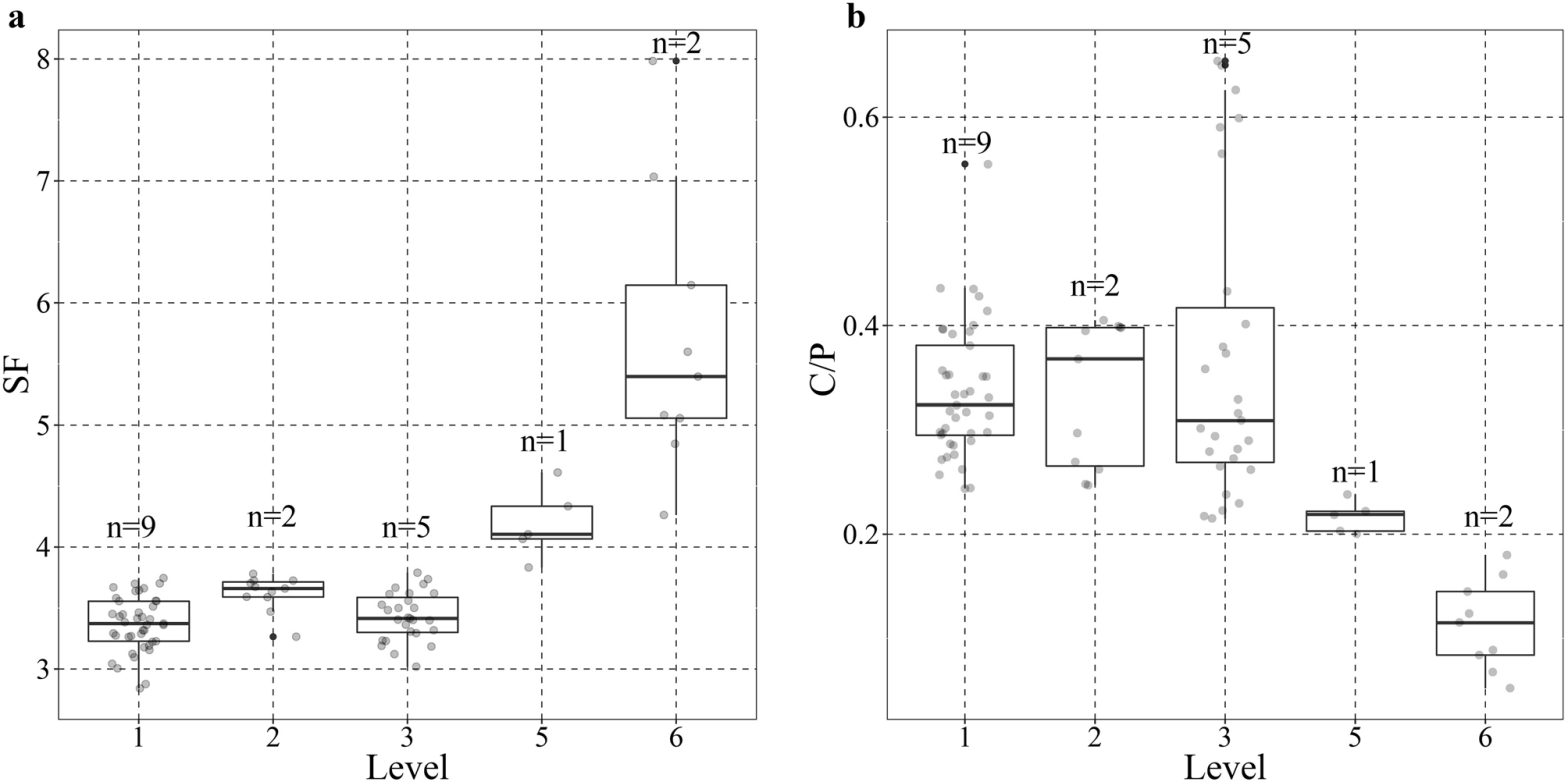
Splitting factor (**a**) and C/P ratio (**b**) results in bone grinding curves by visual level of alteration of the bones^7^. Individual points indicate the value for a single spectrum within a grinding curve, n indicates the number of individual bones analysed for each level.

**Figure 4:**
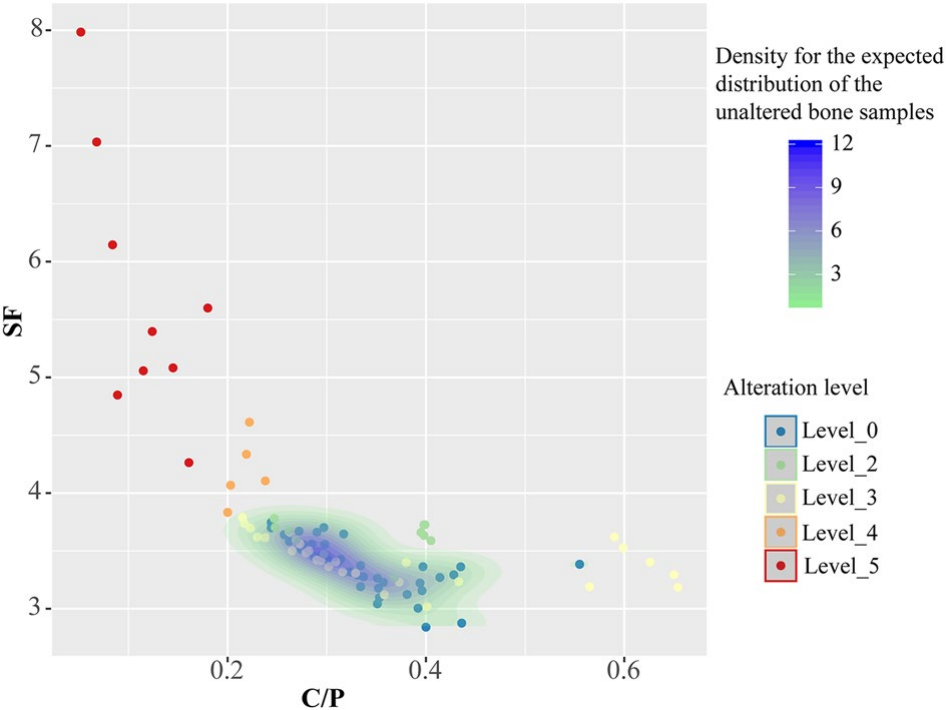
Splitting factor (y-axis) and C/P ratio (x-axis). Points indicate single spectrum and the colour infill indicates the level of alteration according to Stiner et al. 1995^7^. The scale level (green to blue) indicates the distribution intensity of unburnt bones (blue points). Most unburnt bones fall within a limited range of SF and C/P values. Bones with alteration levels 2–3 fall in the same range (with a single exception, probably due to the presence of secondary calcite), but those with levels 5–6 fall outside.

The FTIR results for the bone samples (n = 19) show alteration at different temperatures. Figure 3 shows the results for the splitting factor (SF) and the Carbon/Phosphate (C/P) ratio in bone grinding curves as a function of the visual level of bone alteration defined by Stiner^7^. SF increases with the level of thermal alteration, whereas C/P decreases. Moderately altered bones (alteration levels 2–3) do not differ significantly from unburnt bones (alteration level 0) but highly altered ones (alteration levels 5–6) do. The scatterplot of these variables (Fig. 4) corroborates that bones with alteration levels 5–6 fall outside the predicted range for unburnt bones, whereas bones with alteration levels 2–3 do not (the presence of secondary calcite could explain the higher C/P found in one of these). Repeated grinding increased SF and reduced C/P. The values obtained for bones with alteration levels 5–6 cannot be attributed only to this grinding process, because they are out of the range of the variation produced by the successive grindings (Fig. 3). Localized diagenetic processes may increase the bones’ crystallinity index^37^, but we believe that the parsimonious explanation for this variation is that the bones in layer Xc have been altered at different temperatures, including at least one example of thermal alteration at high temperature (sample ID #15).

#### Energy-dispersive X-ray spectroscopy

Manganese (Mn) was detected in two of the samples used for control: 3% on a deer tooth (sample ID #90), 1.7% to 3% on a long bone (sample ID #91) (Supplementary Fig. S2). These samples come from Praia dos Bifaces, an underground river terrace located ca. 1 km upstream from the Almonda karst spring that undergoes regular winter inundation and in which faunal remains and Acheulean lithics, including bifaces, are found in secondary position^38^. The other control sample is a bone from Aroeira with no indication of manganese coating or of thermo-alteration (sample ID #92). In this sample, only the elements forming carbonated hydroxyapatite (dahllite) were detected.

Calcium (Ca) and phosphorus (P), probably originating from bone, are the most common elements in the seven samples from Aroeira whose colour suggests burning (Supplementary Table S3). Other elements, such as copper (Cu) and iron (Fe), are also present, but in limited proportion (≤ 2.30%). In the case of sample ID #16, high values of aluminium (Al) and silicon (Si), possibly related to the presence of clay remnants, and a very small amount of titanium (Ti), were found on the surface. Manganese was absent (or undetectable) in all seven samples.

### Wood charcoal

#### Description

Charred plant remains were found in four of the 16 samples identified in the field as “charcoal” (Supplementary Table S1) (see Supplementary Text S3 for the description of the non-plant material).

**Figure 5:**
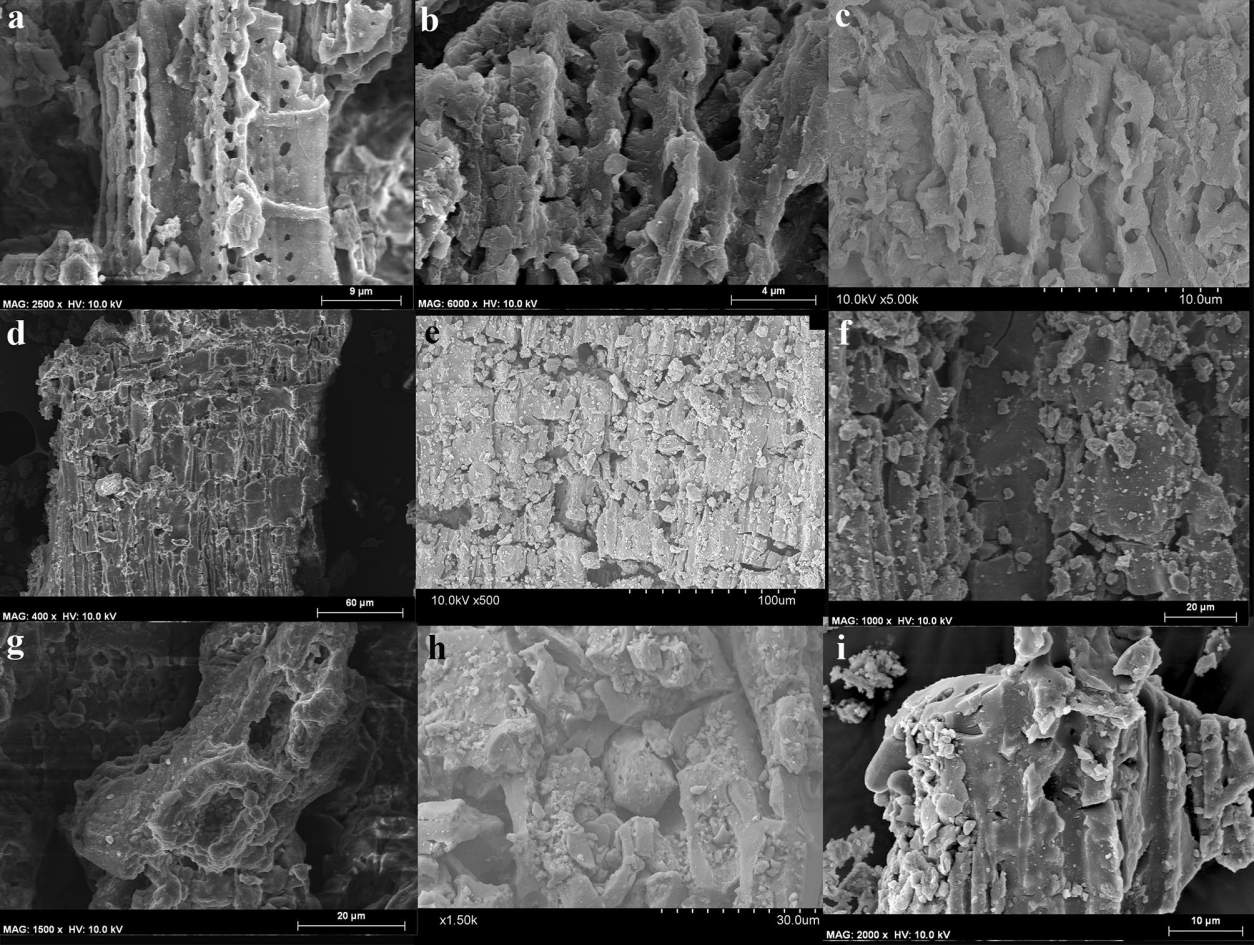
(**a–c**) Plant tissue from sample ID #48. (**d–g**) Sample ID #49; particles are tiny, not reaching 2 mm in length (**d**); only the radial and tangential sections can be observed (**e**–**g**); vessel lumina are 20–30 μm in diameter and display simple perforation plates (**f**); rays are uniseriate and heterogeneous with one row of upright or square marginal cells (**g**). (**h**) Sample ID #51; the plant tissues retain the organic compounds in their interior, as if mineralized rather than carbonized. (**i**) Sample ID#58; cells in anatomical connection.

Sample ID #48 contained a mixture of several organic materials, including plant tissue and poorly preserved bone fragments, all with a burnt appearance. The plant cells are blinded, distorted and in some cases fused together. The xylem anatomy is heterogeneous; it features vessels, rays and fibres but identification beyond the level of dicotyledonous angiosperm is not possible (Fig. 5). Only the cell walls of these plant tissues are preserved; their interior has no organic content, which is characteristic of incomplete combustion.

Samples ID #49, 51 and 58 contained small plant particles (> 2 mm in length) (Fig. 5), too small for the observation of the standard three anatomical sections to be feasible. Based on the preserved vessels and rays, sample ID #49 can be identified as dicotyledonous angiosperm (Fig. 5). In the case of samples ID #51 and #58, it can only be inferred that the plant cells probably are angiosperm tissue in anatomical connection. In samples ID #49 and #51, the interior organic compounds are retained (Fig. 5), which could result from mineralization or the curtailment of combustion prior to the gasification stage.

#### Elemental composition

Two samples (samples ID #48 and #49) were analysed by Energy Dispersive X-ray. In sample ID #48, two fiber cell walls and a conductive vessel were measured in the longitudinal plane of the wood. All show high carbon (C) and oxygen (O) content, both by weight and atomic % (Supplementary Fig. S3). Similar values were obtained for the fibre cell walls, parenchymal ray and a conductive tissue vessel in sample ID #49. These data are indicative of incomplete combustion. Calcium (Ca), silicon (Si), magnesium (Mg), phosphorus (P) and potassium (K) are present in small percentages; among these elements, Ca is the most abundant (Supplementary Fig. S3).

The C and O values of the two plant samples are consistent with the elemental composition of wood, of which they are its two basic constituents^39^. The values of Mg, P and K are within the limits established by Ragland^40^ for wood, their variation possibly reflecting the fact that each sample comes from a different type of plant. It is also possible that the O, Ca and Si values reflect a post-depositional mineralization process in which oxalates and silicates are by-products of the chemical or biological degradation of organic matter.

The elemental composition of the samples is like that for Palaeolithic charcoal burnt at > 300 °C^41,42^. There can be little question that samples ID #48 and #49 represent combusted material, but the analysis does not allow us to determine whether the fire that caused the combustion was anthropogenic or natural.

### Sediments

#### Micromorphology

Thin sections were cut from a sediment column extracted as three contiguous blocks (samples ID #60–62) across the thickness of the deposit in which the burnt material was retrieved (Supplementary Fig. S4 and Table S4). Their micromorphological analysis reveals a complex sediment devoid of direct evidence for in-situ combustion.

**Figure 6:**
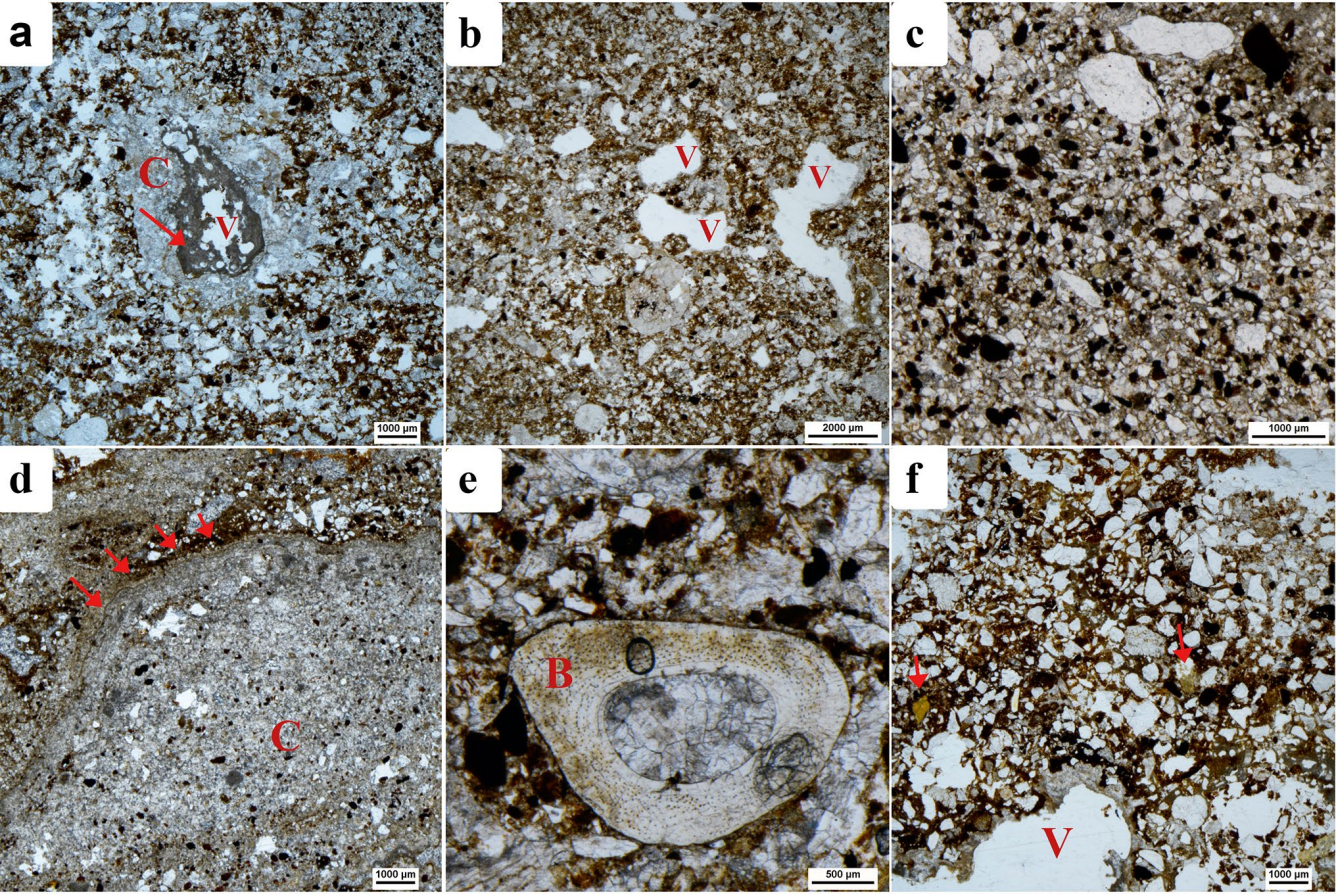
Microphotographs showing the characteristics of the main microfacies detected in sample ID # 60 where the combustion residues are affected by water action. (**a**) Microfacies 2; matrix composed of ‘clotted’ calcite (C) with sparitic calcite formed by dripping water infilling voids (V) (indicated by red arrow). PPL. (**b**) Microfacies 3; modified planar and complex voids (V). PPL. (**c**) Microfacies 3; charcoal dots dispersed in the matrix. PPL. **(d)** Microfacies 4; laminated coating above the cemented calcite (indicated by red arrows). PPL. (**e**) Microfacies 5; sub-rounded bone fragment (B), pale brown in PPL. (**f**) Microfacies 8; phosphate nodules from degraded coprolite fragments (indicated by red arrows) and sub-rounded vesicle (V) indicative of cold conditions. PPL.

Twelve microfacies in gradual, smooth transition have been recognized (Supplementary Table S4). Representative features of these microfacies are shown in Fig. 6. The sequence of microfacies shows a fine-graded bedding, from a very fine (M1) matrix at the base of the column to a much coarser, homogeneously sand-sized, limpid matrix at the very top (M12). The latter features a generally laminated arrangement of the constituents, namely bone micro-fragments (µm- to mm-sized), which, like some cm-sized subangular quartz and limestone block fragments, are mostly subangular.

Mesofauna-related bioturbation is observable throughout. Modified planar and complex voids are frequent. Examples (mm- and cm-sized) can be found in samples ID #60 and 62, the latter from the very top of the column, in microfacies M12, which is associated with the ageing of earthworm excrements. The voids are deformed and strongly coalesced with the presence of thin, dark brown coatings of fine material probably the result of soaking. Bioturbation related to the presence of roots is also noticeable, with voids infilled by post-depositional secondary sparitic calcite. Typical hypocoatings of sparitic calcite are present throughout, and are more apparent in microfacies 4, 5, 6, 8 and 12. Similarly, sparitic limestone fragments together with light gray sparitic calcite are observable in sample ID #60, although the calcitic material presents neither parallel orientation nor rhomboid calcite crystal pseudomorphs. This suggests that even if ash had originally been present, none would have been preserved. An eluvial horizon has formed at the top of the column, at the contact with sample ID #60, where sparitic calcite is most abundant.

Scattered phosphatic features, including sub-rounded micro-fragments of carnivore coprolites and apatite nodules (µm-sized, related to bone recrystallization), are observed throughout.

Note that calcined, burnt and unburnt bone material (white, orange/brown/black and pale yellow in plane polarized light—PPL) occurs frequently only in the microfacies defined in sample ID #60 (Supplementary Fig. S4). Specifically, a concentration of burnt and calcined bones is found within a massive reddish clay groundmass restricted to the block’s upper area. Whether the clay is burnt cannot be determined by micromorphology, though. Iron-manganese nodules (µm-sized) together with frequent dark flecks are present throughout, except in microfacies M11 and M12 of sample ID #62. They are natural constituents of the sediment, not a by-product of thermal alteration. No charcoal cell structures were detected, but charcoal dust is possibly present in samples ID # 60–61. Phytoliths and other vegetal remains were not observed.

#### Organic chemistry

Two samples of sediment were analysed to detect combustion traces and assess the potential presence of plant material inputs and organic matter degradation. The lipid profile presented by sample ID #63 (layer Xc) includes short- and medium-chain *n*-alkanes. The carbon number distribution of the plant wax-derived *n*-alkanes ranges from C_12_ to C_25_ with a predominance of higher concentrations in the odd C-numbers although even C-numbers are also present (C_12_ and C_14_). C_25_ is the most abundant with a concentration of 0.9 µg/g of dry sediment. No polycyclic aromatic hydrocarbons (PAHs, organic compounds that are produced during the combustion of organic material) were detected (Supplementary Fig. S5).

In sample ID #64 (layer XI), the lipid profile includes medium- and long-chain *n*-alkanes. The carbon number distribution of the plant wax-derived *n*-alkanes ranges from C_17_ to C_33_ with a predominance of higher concentrations in the odd C-numbers although even C-numbers are also present (C_28_, C_30_ and C_32_). C_31_ is the most abundant with a concentration of 0.8 µg/g of dry sediment. No PAHs were detected (Supplementary Fig. S5).

Previous studies conducted on vascular plants^43–45^ differentiate between terrestrial taxa (i.e. the xero-mesophilic group), characterized mainly by *n*-alkanes maximizing at C_29_ and C_31_, and submerged living taxa (i.e. the meso-hygrophilic group), maximizing at n-C_25_ and n-C_27_. Moreover, vascular plants are characterized by a thick leaf-wax layer producing a wide range of *n*-alkanes ranging from C_31_ to C_35_ while mosses and ferns produce only a small amount of *n*-alkanes, with C_17_, C_23_ and C_25_ being the most common carbon numbers present^46,47^. Finally, green and red algae are rich in C_15_ and C_17_ *n-*alkanes^48^.

The presence of even C-number alkanes in terrestrial and aquatic plants is uncommon. When present in sediments, they usually relate to the natural degradation of bacterial, microbial and algal detritus and their microbial or geochemical alteration^49^.

Our *n*-alkane results are consistent with a submerged/emergent plant origin (e.g., mosses and algae) in all sediment samples. The wet-habitat taxa thus probably reflect the percolation of water, which is also observed at the microscopic scale. Interestingly, the presence of terrestrial vascular plants is only observed in sample ID #64 from layer XI, the medium-chain *n*-alkanes suggesting the presence of cellulose and lignin-rich plant remains (i.e., leaves, branches, twigs) in this sample. No PAHs were detected, meaning that no in-situ thermo-alteration of the sediment occurred or that, because of the water action, the compounds have been completely degraded.

#### FTIR

The FTIR results for the loose sediment samples (n = 13) show there is no evidence of anthropogenic calcite and we have detected no thermally altered clay in the samples from layer Xc. The main mineral components of the sediments analysed are calcite, clay, quartz, and, to a lesser extent, dahllite. Some samples show a limited presence of calcite or no calcite at all, so the distribution of calcium carbonate is far from homogeneous. The only sample that showed a different spectrum is the black nodule from layer XI (sample ID #89), composed of haematite.

#### Magnetism

The sediment samples are more magnetic than the lithic samples (see below), but present considerable variation. They are dominated by magnetite (T_*C*_ = 580 °C; Supplementary Fig. S6). However, a variation of one order of magnitude is observed between the samples collected close to the potentially thermally altered area (Supplementary Fig. S6a) and most of the unheated control samples which are around 10 times weaker (Supplementary Fig. S6b). Two out of the six control samples display magnetization values of the same order as the potentially heated ones (Supplementary Table S5), showing that even control samples are highly variable. These results suggest that some type of geological process homogenized the magnetic properties of the sediments.

### Lithics

**Figure 7:**
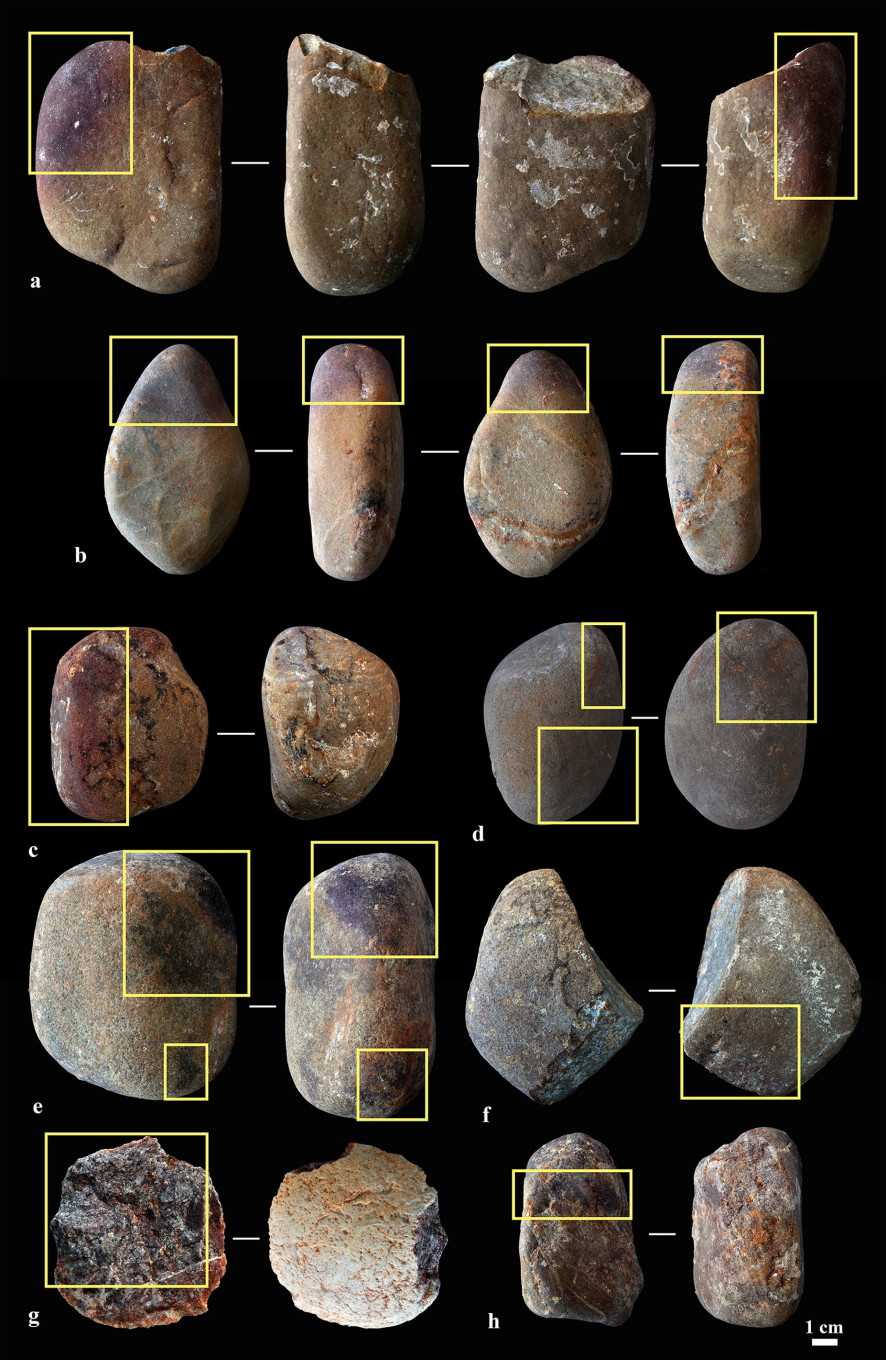
Quartzite artefacts (layer X) with reddish and blackish areas (marked with rectangles) indicative of possible thermal alteration (**a** Sample ID #104; **b** Sample ID #105; **c** Sample ID #106; **d** Sample ID #107; **e** Sample ID #108; **f** Sample ID #109; **g** Sample ID #110; **h** Sample ID #111).

Among the lithics, we identified eight items presenting a colour that might result from thermal alteration (Fig. 7)^50^. To assess whether such was indeed the case, we analysed the magnetic properties of two (samples ID #104–105; the first presents an intense reddish colour at one end) (Supplementary Table S5). Additionally, cobbles representing three types of local quartzites that are also represented at the site (samples ID #101–103) (Supplementary Fig. S7) were analysed under controlled temperature conditions to study how their magnetic properties reacted to heating.

**Figure 8:**
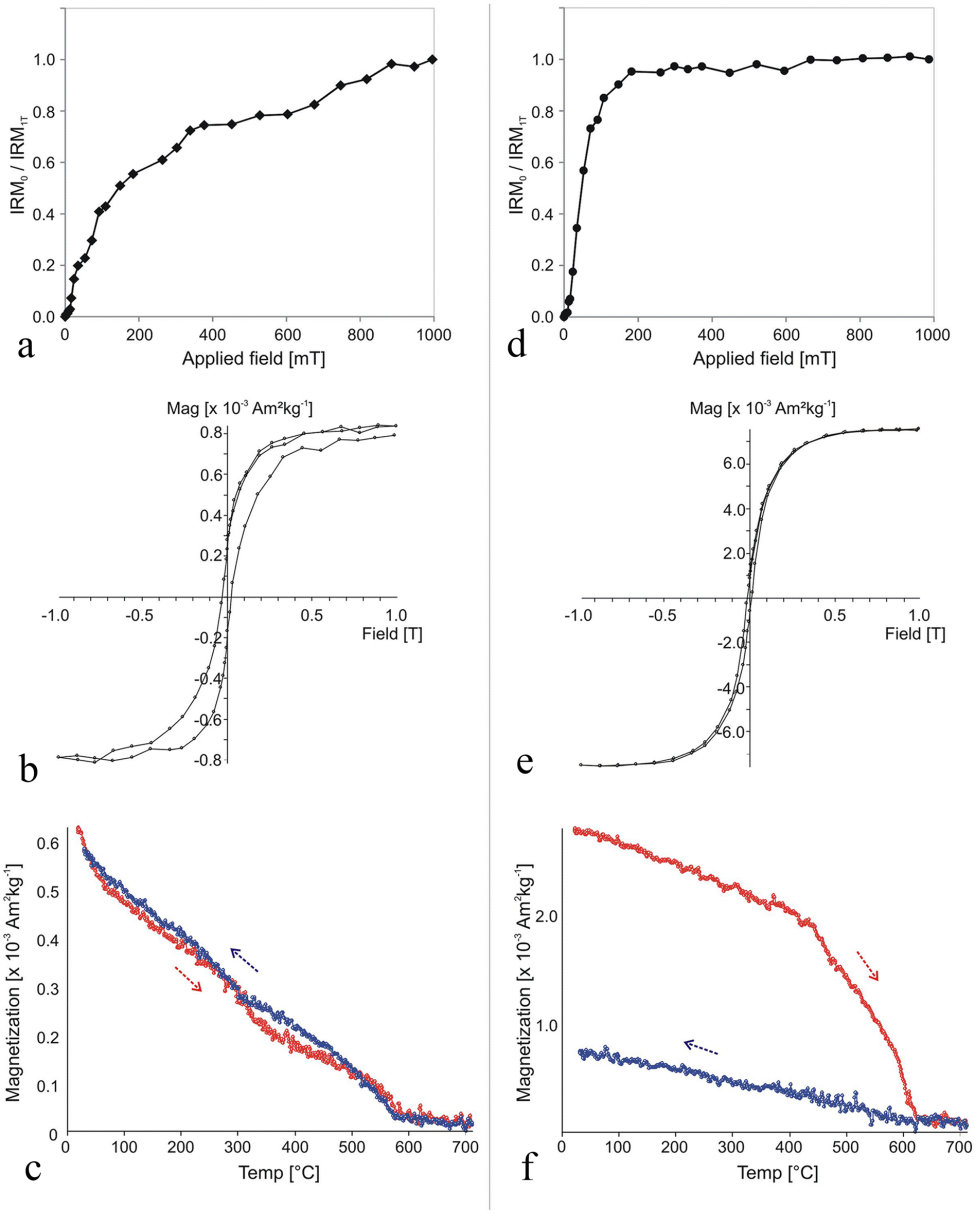
Comparison of the magnetic properties of two subsamples of archaeological quartzite cobble (sample ID #104), reddened at one end, possibly the result of heating. (**a–c**) Sample ID #104_3 (unheated sample); (**d–f**) Sample ID #104_2d (possibly heated subsample from reddened end). **a**,**d** Normalized progressive IRM acquisition curves. **b**,**e** Hysteresis cycles. **c**,**f** Thermomagnetic curves (magnetization vs. temperature). Heating (cooling) cycles are indicated for each sample.

No significant differences were found between the magnetic properties of the archaeological quartzites and their experimental counterparts, except for the reddened cobble, which shows a concentration of magnetic mineral one order of magnitude higher, suggesting heating (Supplementary Table S5). This cobble (sample ID #104) also displayed more internal variation than the other (sample ID #105), which presents thermomagnetic curves with similar room temperature magnetization values (J_30_) and a range of variation of subsamples of the same order of magnitude as the unheated experimental control samples at room temperature (Supplementary Table S5). In contrast, the subsamples from the unheated and the potentially heated areas of reddened cobble (sample ID #104) show very different magnetic properties. The unheated subsample contains haematite as its main remanence carrier with isothermal remanent magnetization (IRM) curves unsaturated at 1 T, wasp-waisted hysteresis loops and a phase with a Curie temperature (T_*C*_) of around 675 °C (Fig. 8a–c) (T_*C*_ is the specific temperature of every ferromagnetic *s.l.* mineral above which the ability to retain a remanent magnetization is lost; e.g., the T*c* of magnetite is 580 °C, that of haematite is 675 °C^51^). By contrast, the potentially heated subsample is dominated by a low coercivity mineral with an IRM curve almost saturated around 200 mT and a soft hysteresis cycle (Fig. 8d,e); the main carrier identified is a phase with a T_C_ slightly over 600 °C, probably maghaemite (Fig. 8f). This thermomagnetic curve is almost one order of magnitude more magnetic than that of the unheated subsample, suggesting exposure to heating.

The results obtained for the three types of experimentally heated quartzite cobbles (samples ID #101–103) indicate that heating up to 300 °C and even 600 °C barely increases their original (room temperature) magnetization intensities (Supplementary Fig. S8 and Supplementary Table S5), with sample ID #102 actually showing a small reduction in magnetization when heated to 300 ºC (as a result of the neoformation of haematite). Even though the dominant original diamagnetic nature of both the archaeological and the experimental quartzites clearly limits the creation of new ferromagnetic minerals, the magnetic enhancement reflected by the potentially heated subsample of the reddened cobble (sample ID #104_2d) clearly exceeds that observed in the experimentally heated samples (Supplementary Fig. S8).

### Spatial distribution of the burnt finds

**Figure 9:**
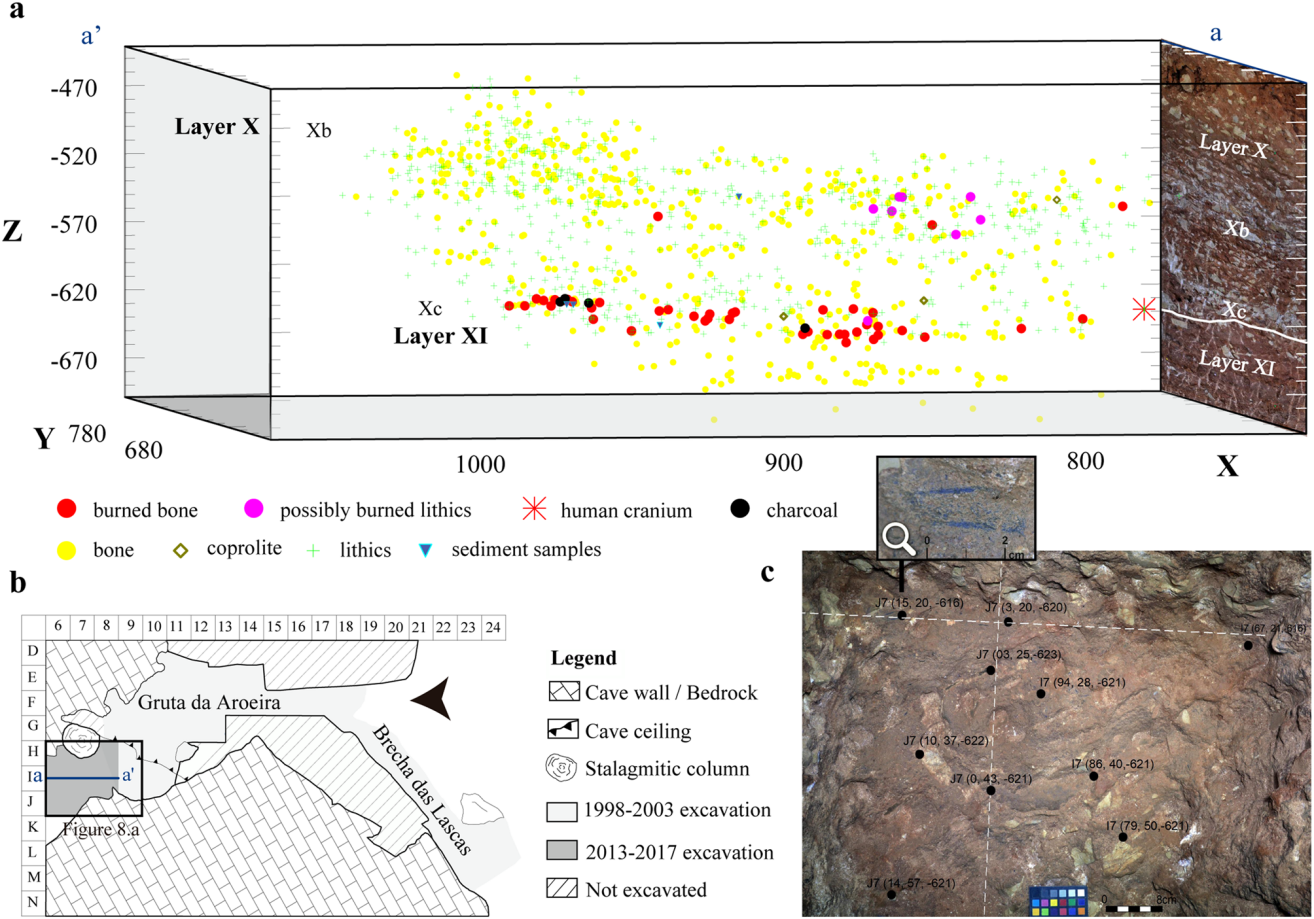
(**a**) Distribution of the burnt remains in layer X of Gruta da Aroeira. (**b**) Site plan with location of the excavation trench. (**c**) The exposed surface of unit Xc; the black dotes denote burnt items and the inset zooms-in on a burnt bone in square J7.

The vertical distribution of the Layer X burnt remains suggests two discrete accumulations. Even though more numerous towards the base of the unit (Xc) (Fig. 9), three burnt bones were also found higher-up in Xb, alongside six lithic items bearing reddish and blackish areas indicative of possible thermal alteration, among which the previously described reddened cobble (sample ID #104).

## Discussion

### The combustion-related elements

The thermal alteration of bones, corroborated by taphonomic analysis, XEDS and FTIR, provides direct evidence that combustion-related elements are indeed present at Gruta da Aroeira. Although manganese is sporadically found, the analyses rule out that manganese oxide is responsible for the bones’ colour, and the same for copper and iron. FTIR confirms that the burnt bones were altered at high temperatures, with the calcined ones (grades 5 and 6; 14% and 9%, respectively) implying exposure to fires above 650 °C. The data also show that the bones were variably exposed to thermal alteration: those presenting a black coloration (lower levels of alteration) have SF and CP values like those of the unburnt control samples, while those with higher grades of alteration (calcined) burned at both high- and low-temperatures and in some cases only partly.

Wood charcoal has been positively identified and bears alterations similar to those identified in remains from Palaeolithic sites in which the anthropogenic nature of the fire evidence is undisputed^52–55^. Namely, the elemental composition of the remains is like that found at Bolomor^41^, Abrigo de la Quebrada and Cova de les Cendres^42^, and shows that the wood was subjected to a combustion process that reached the ignition phase and, as shown by the C content, stopped at around 300–400 °C, resulting in charcoal.

The soil micromorphology samples present many of the characteristics of the remains of disturbed fires subject to various geological processes^56^. As exemplified in the Middle Palaeolithic of Kebara (Israel)^57^ and Hohle Fels (Germany)^58^, leaching leads to calcitic ash being rapidly dissolved and washed out of the system, while phytoliths are likely to be dissolved at high rates of water washing of the sediments^59^, explaining why thin sections show none. Undisturbed fires, in contrast, tend to present a microstratigraphy that comprises an uppermost ash-rich lens overlying a charcoal-rich layer, which in turn overlies thermally altered substrates^60,61^. This stratigraphic arrangement is not observed in our samples, which might result from either pedogenetic removal and transformation of the evidence or from post-depositional scattering of the original fire context.

The magnetic properties of the reddened quartzite cobble (sample ID #104) and the small thermoaltered bones found in Xc (sample ID #60), the main concentration of burnt material, are consistent with both mechanisms, while the presence of *n*-alkanes related to mosses and ferns indicates exposure to the effects of post-depositional degradation due to water action. This factor could also explain the absence of PAHs, which have a higher solubility than alkanes^62^. However, FTIR failed to detect thermally altered clay in the sediment samples from the same area and unit, and the magnetic properties of those samples show that they were subject to a process whereby heated and non-heated material was homogenised. Thus, the evidence pleads in favour of the cluster of burnt materials found in layers Xb and Xc of Gruta da Aroeira to represent, primarily, the outcome of a mechanical process—the post-depositional scattering of a combustion feature. Chemical processes subsequently produced additional in-situ alteration of some of the fire-related constituents originally present.

### The origin of the fire

Temperature is an unreliable criterion to discriminate between wildfires and fires managed by hominins because heat can reach between 200 °C and 800 °C in both cases^63,64^. A wildfire catching on a bush or tree reaches very high temperatures for a few minutes and can char any bones exposed on the ground^64^. However, the burning damage observed on carcasses exposed to natural landscape fires is very different from the pattern observed in layer X of Gruta da Aroeira. Experimental studies have shown that bones exposed to natural fires have highly variable alterations and, in general, show damage that is both very slight and circumscribed to the exposed side and to some anatomical parts (those farthest from the ground as well as ridges, condyles, edges or epiphyses^64^), which can become carbonized or present a brownish appearance with superficial cracking and turn brittle^65^; very few, however, reach the calcination stage. The bones from Gruta da Aroeira described here display thermal alteration on all sides and include calcined specimens that were subject to very high temperatures, which is consistent with burning under a concentrated focus of heat, such as a campfire, rather than in a wildfire.

Aside from brittleness related to heat exposure, the Gruta da Aroeira burnt bones are zooarchaeologically like the unburnt ones: both assemblages, burnt and unburnt, show the same high degree of breakage and are mainly composed of non-identifiable material half of which corresponds to medium-sized ungulates. These features indicate that the faunal assemblage as a whole is mostly anthropogenic and that its burnt and unburnt constituents underwent the same kinds of biostratinomic and post-depositional processes—bioturbation, dissolution, cryoturbation, carnivore activity, reworking by water and gravitational processes, among others^6,66–70^. Put another way, there is every reason to think that both have the same origin and none to believe otherwise.

This interpretation is fully consistent with everything we know about site formation at Gruta da Aroeira. Gravitational processes are the main mechanism of accumulation of layer X as exposed in the excavated area, located deep inside of the original cave entrance. At present, our trench is around 12 m from where the cave fill was erosionally cut by the receding slope. At the time of occupation, however, the inhabited cave porch must have been located even farther out, as demonstrated by the outcrops preserved in the current exterior locus of the site, the so-called Brecha das Lascas^71^. In our excavation trench the stratification tends to horizontal because it lies at the foot of a talus whose dip is, however, significant. Like everything else—stone tools^50^, cut-marked or otherwise human-modified bones, or the Aroeira 1–3 human fossils (cranium and teeth)^36,72^—the burnt materials found in our trench must have been syn-depositionally displaced along the talus from a primary locus of deposition located upslope, nearer the cave porch—as proposed for levels 15–19 of Gruta da Oliveira, a Middle Palaeolithic cave site located ca. 40 m lower down in the Almonda escarpment^73^.

In the area of our trench, the Gruta da Aroeira deposit is sealed by thick flowstone, dated to between 326.4 ± 13.4 ka (2σ), at the top, and 417.7/ + 37.3/−27.5 ka (2σ), at the base, which implies a rather constant supply of dripping water for a period of anywhere between at least 50,000 and possibly as much as 140,000 years^36,71^. That this water also percolated through the underlying open work, predominantly clast-supported deposit, where it eventually precipitated, is shown by the U-Th ages obtained for calcite crystals coating sedimentary voids found below the flowstone^36^. The *n*-alkane results reported here also reflect the operation of these processes. For instance, the presence of mosses and algae in all the sediment samples indicates a soaked burial environment that could also have included material from the massif’s plant cover transported into the cave by the flowstone-forming dripping water. Conversely, these mechanisms necessarily imply that ash, phytoliths or hydrocarbures would have been leached and become undetectable at analysis—as indeed is the case.

Our data do not allow us to define the exact nature of the hominin activities responsible for the production of the thermally altered remains (bones and cobbles). Calcination implies direct exposure to heat and flames^7,60^. The higher degrees of burning could be caused by proximity to the focus of the combustion^74^. A likely scenario for the production of the observed patterns is one whereby bones and cobbles previously discarded were accidentally burnt by the lighting of hearths in combination with the tossing of food refuse into the fire.

The fact that hearths are rarely found in other Acheulean contexts remains in need of additional investigation. The few examples mostly relate to residential camp site types, independent of latitude or setting (i.e., whether open-air or cave), e,g. Qesem^19,75^ or Beeches Pit^26^. Yet, at other residential sites, e.g. Caune de l’Arago^76^ or Atapuerca^77^, fire is strikingly scarce, if not altogether absent. Therefore, factors other than site function, such as tool-making technology, subsistence strategy, animal exploitation patterns, paleoclimatic background, seasonality of the occupations, social behaviour, or intra-site variability may have also played a role in the production of this contrast.

## Conclusions

Acheulean layer X of Gruta da Aroeira, dated to around 400 ka, contributes new data on the controlled use of fire and human behaviour among the Middle Pleistocene populations of south-western Europe. By-products of combustion, namely burnt bones (some of which were likely exposed to high temperatures), charcoal, possibly heat-altered cobbles and sediments (revealed by the modification of their magnetic properties) have been identified. Coupled with the effects of post-depositional leaching, the fact that the clusters of such by-products found at excavation reflect minor syn-depositional displacement from a primary context of occupation located nearer the cave porch explain the lack of ash and of in-situ sediment rubefaction.

When fireplaces are preserved intact, in-situ fire use is self-evident and requires no additional proof. In most Palaeolithic sites, however, preservation to such a degree is a rare event. To assess whether fire was used in a controlled manner, a multi-analytic approach is required. Otherwise, the absence of evidence can easily be mistaken for an evidence of absence. As we hopefully have been able to demonstrate here, the patchy record of fire in the Lower Palaeolithic has to be seen in light of preservation issues, not just site function or hominin abilities. This is especially the case when dealing with cave sites but also applies to open air situations, where the occurrence of wildfires additionally complicates interpretation. At Gruta da Aroeira, the multi-analytic approach we advocate revealed that burnt remains occurred in two different horizons, strengthening the case for their anthropogenic origin and for use of fire at the site to have been part of a behavioural routine rather than a one-off occurrence.

## Materials and methods

The assemblage analysed here comes from fieldwork undertaken between 2013 and 2017. A total of 43 burnt bone remains recovered from the layer X were analysed in this study. The damage attributable to burning was described by colour (naked eye) using the six-grade scale proposed by Stiner^7^: (1) slightly burned, (2) more than half carbonized, (3) fully carbonized, (4) slightly calcined, (5) more than half calcined and (6) fully calcined (completely white in colour). The anatomical identification of the charred remains was carried out using an optical microscope. Elemental analyses using energy dispersive X-ray were carried out on two plant samples. Micromorphology analysis was conducted on four thin sections prepared from three undisturbed sediment blocks. Loose sediment and bone samples were collected for Fourier transform infrared spectroscopy (FTIR) analysis. Energy-dispersive X-ray spectroscopy of burned bones were analysed. Magnetic properties of several archaeological samples were analysed. Additionally, pebbles representing three types of local quartzite, but similar to that found at the site, were heated under controlled temperature conditions so as to analyse any variation in their respective magnetic properties. Additional details on the methods used to analyse the samples are provided in Supplementary Text S2.

## Supplementary information

Supplementary information
