# Supplementary material for: Early evidence of fire in south-western Europe: the Acheulean site of Gruta da Aroeira (Torres Novas, Portugal)
Source: Sci Rep. 2020 Jul 21;10:12053. doi: 10.1038/s41598-020-68839-w (PMC7374095; doi:10.1038/s41598-020-68839-w)
Supplement: Supplementary file 1 — Supplementary information [file 41598_2020_68839_MOESM1_ESM.pdf]

Early evidence of fire in south-western Europe: the Acheulean site of Gruta da Aroeira (Torres Novas, Portugal)

Montserrat Sanz[1,2,*], Joan Daura[1,2], Dan Cabanes[3], Natalia Égüez[4], Ángel Carrancho[5], Ernestina Badal[6], Pedro Souto[7], Filipa Rodrigues[2], João Zilhão[2,8-9]

[1]Grup de Recerca del Quaternari (GRQ-SERP), Departament d'Història i Arqueologia, Universitat de Barcelona, Carrer Montalegre, 6, 08001 Barcelona, Spain

[2]UNIARQ-Centro de Arqueologia da Universidade de Lisboa, Faculdade de Letras. Universidade de Lisboa, 1600-214 Lisbon, Portugal.

[3]Department of Anthropology, Rutgers University, Biological Sciences Building, 32 Bishop Street, New Brunswick, NJ 08901, USA

[4]Archaeological Micromorphology and Biomarkers - AMBI Lab, Instituto Universitario de Bio-Orgánica Antonio González. Universidad de La Laguna, 38206 Tenerife, Spain.

[5]Área de Prehistoria. Dpto. Historia, Geografía y Comunicación. Edificio I+D+i. Universidad de Burgos. Plaza Misael Bañuelos s/n. 09001 Burgos, Spain.

[6]PREMEDOC-GIUV2015-213, Departament de Prehistòria, Arqueologia i Història Antiga, Universitat de València, Av. Blasco Ibañez 28, 46010 Valencia, Spain

[7]STEA - Sociedade Torrejana de Espeleologia e Arqueologia, Quinta da Lezíria, 2350-510 Torres Novas, Portugal.

[8]Departament d'Història i Arqueologia. Universitat de Barcelona, Carrer Montalegre, 6, 08001 Barcelona, Spain

[9]Institució Catalana de Recerca i Estudis Avançats (ICREA), Passeig Lluís Companys 23, 08010 Barcelona, Spain

*Corresponding author: montsesanzborras@ub.edu

SUPPLEMENTARY TEXT 1

1. Site description

The Gruta da Aroeira (39° 30' 20" N; 08° 36' 57" W) forms part of the Almonda karst system and is located in the Central Limestone Massif of Estremadura in the municipality of Torres Novas (Santarém, Portugal). The cave is at 139 m a.s.l. and corresponds to an ancient outlet of the river Almonda (Supplementary Fig. S1).

The site was speleologically discovered by STEA (*Sociedade Torrejana de Espeleologia e Arqueologia*) in 1991, when explorations identified a network of passages (*Galerias Pesadas*) sealed by a cone of sediments containing Middle Pleistocene faunal remains and artefacts[1]. *Galerias Pesadas* was, in fact, the name erroneously used in the first fieldwork (1998-2002) undertaken on the sedimentary fills exposed in both the cliff face and the reopened cave entrance[2]; this designation actually corresponds to the interior conduits of the karst system. The designation given in 1991 to the reopened cave entrance excavated 1997-2002 and 2013-2017 is Gruta da Aroeira.

In 2013, archaeological work resumed. The focus was on excavating and sampling the area at the back of the cave, corresponding to grid squares H6 to H8, I6 to I8, and J6 to J8 (Figure 9 and Supplementary Fig. S1), with the aim of reaching bedrock and establishing the time depth of the archaeological deposit, for which previously dated speleothem samples provided a minimum age of ~400 ka[3]. At the back of the cave, the stratigraphic sequence comprises three major units with an overall thickness of 4 m. The uppermost unit, Unit 1, is a brecciated infill capped by flowstone dated to 44.8±2.0 ka (2σ). Unit 2 is a 2.2-m thick mud-supported breccia, rich in angular and sub-rounded clasts, corresponding to Acheulean layers X-Xb/c (which are the upper and lower parts of a single layer, excavated in 1998–2002 and 2013–2017, respectively), and is capped by a second flowstone whose base dates to 418/+37/−27 ka (2σ). Unit Xc, which yielded most of the fire evidence, is composed mainly of silty sand with dispersed gravel (Figures 9 and Supplementary Fig. S1). The basal unit, Unit 3, is an endokarst fluvial deposit, comprising two layers: XI is a 0.4 m-thick silty sand with scattered gravel and faunal remains but no artefacts, and XII is a 0.5 m-thick, archaeologically sterile, slightly gravelly sand. The external layer of a stalagmite column, which, based on available cross-sectional views, seems to have grown from the top of layer XII and to have been buried by subsequent infilling (Units 1 and 2), dates to 406±30 ka (2σ), providing a maximum

age for layer X, the deposition of which can, therefore, be dated to the 390-436 ka interval[4].

The archaeological remains retrieved in layer Xb/c are mostly lithic artefacts and faunal remains. It also yielded the human cranium Aroeira 3. The faunal remains are highly fragmented, consisting primarily of isolated teeth, phalanges, carpal/tarsal bones, and antler fragments. Among the faunal remains from layer Xb/c, cervids[5] and equids predominate. Rarer species include Rhinocerotidae (likely *Stephanorhinus* cf. *hundsheimensis*), bear (*Ursus* sp.), as well as large bovid, caprids (Caprinae), tortoise (*Testudo* sp.) and a macaque (*M. sylvanus* cf. *pliocena*)[6].

SUPPLEMENTARY TEXT 2

2. Materials and Methods

2.1. Excavation methodology

The current study uses the finds made during the fieldwork undertaken between 2013 and 2017. The material collected 1998–2002 is not included[2,7]. The position of all finds was recorded in three-dimensions within a 1 m^2 grid system, identified by letter and number. Small items and fragments were bagged by 1 m^2 units of provenience.

Excavation used a demolition hammer, as both archaeological and palaeontological remains were completely encased in rock-hard carbonate breccias. Lithics and bones were subsequently extracted from the rock using a pneumatic micro-hammer and micro-chisel (Mod. CTS 178) and normal air scribe (Mod. W 224). After cleaning, bone fragments were joined and a final thin layer of consolidant (Paraloid B-72), dissolved in acetone, was applied to the entire surface. The burnt remains were not consolidated.

2.2. Zooarchaeological and taphonomic analyses

A total of 43 burnt bone remains recovered from layer Xb/c were analysed in this study (Supplementary Table S1). The zooarchaeological analysis involved identification of animal bones to genus or species level where possible. The anatomical region of origin of the bone remains (cranial, axial and appendicular) was determined, along with the skeletal element, portion of element, side and age (infantile, juvenile, adult or senile), following standard zooarchaeological methods[8,9]. Non-identifiable fragments were assigned to broader anatomical categories in relation to their skeletal tissue (e.g. flat, long or spongy bone) and body-size categories (e.g. small mammal or medium ungulate).

To determine the effects of biostratinomical and post-depositional processes, bones were examined following standard criteria[10,11], although taphonomic analyses focused primarily on the thermal alteration of bone surfaces. The damage attributable to burning was described by colour (naked eye) using the six-grade scale proposed by Stiner[12]: (1) slightly burned, (2) more than half carbonized, (3) fully carbonized, (4) slightly calcined, (5) more than half calcined and (6) fully calcined (completely white in colour). Burned bone surfaces were analysed at the macroscopic level under a binocular device (NIKON

SMZ800) housed at the *Centro de Investigación UCM-ISCIII sobre la Evolución y Comportamiento Humanos* in Madrid.

2.3. FTIR

A total of 13 loose sediment samples (samples ID #77-89) and 19 bone samples were collected for Fourier transform infrared spectroscopy (FTIR) analysis from layers F, Xb, Xc and XI (Supplementary Table S1). Thermal alteration in bone samples was determined visually and the bones were classified in line with Stiner[12]. In addition, four sediment samples associated with burnt bones were analysed.

The analyses were performed using a Thermo Scientific Nicolet iS5 FT-IR spectrometer in the Laboratory for MicroArchaeology in the Department of Anthropology (ALMA) at Rutgers University. Approximately 1 mg of each sample was ground and mixed with 80 mg of KBr in an agate mill. Infrared spectra were obtained by scanning the samples 32 times at 4 cm^{-1}. In the sediment samples, the geogenic or anthropogenic (i.e., ash) origin of calcite was determined in line with Regev and Poduska[13,14], and clays exposed to high temperatures were identified using specific absorptions in the clay spectrum[15]. In the bone samples, the crystallinity index was calculated using the splitting factor (SF) of bone in the FTIR spectra in line with Weiner and Bar-Yosef[16] and the C/P ratio was estimated in line with Wright and Schwarcz[17]. Since the grinding intensity employed can affect the SF [18], we used grinding curves to address this issue. To obtain these curves, a small bone fragment was first gently ground to produce a pellet. Once the initial spectrum had been recorded, the pellet was returned to the mortar, lightly ground, to produce a new pellet, and a new measurement taken. Each grinding produced a slightly different SF and narrower peaks, indicating that as the particle size was reduced, absorbance in the spectra increased. This operation was repeated until the spectrum obtained had a noisy aspect and measurements were no longer reliable.

2.4. Energy-dispersive X-ray spectroscopy

A JEOL JSM-7600F SEM was used in the scanning microscopy studies. An INCA OXFORD detector for X-ray energy dispersive spectroscopy, XEDS, was attached to the microscope, which was operated at 15 kV and at a 10 mm working distance. These

analyses were carried out at the ICTS laboratory of the *Centro Nacional de Microscopía Electrónica* (*Universidad Complutense de Madrid*).

The specimens were first inspected visually to define areas of interest, from which three or four very small grains were obtained by gently scratching the surface. The grains were placed on carbon tape and attached to the metallic sample holder. The composition of each grain was analysed at several points.

The results of the X-ray diffraction pattern obtained on seven burned bone samples are shown in Supplementary Table S3. To assess test validity and result reliability, three additional controls were used. Thus, we tested manganese coating on two additional samples – a deer tooth (sample ID # 90) and a long bone (sample ID # 91) (Supplementary Table S1 and Supplementary Fig. S2) – recovered from Praia das Bifaces (Entrada do Vale da Serra) in the same network of the Almonda karst system[1]. The third sample was a bone from layer X that presented no thermal alteration (sample ID #92) (Supplementary Table S1 and Supplementary Fig. S2). All three controls were treated in exactly the same way as the specimens described above.

2.5. Wood charcoal

The identification of wood material with the naked eye was not feasible during fieldwork; thus, 16 small samples were collected based on their dark, grey or black appearance (Supplementary Table S1: samples ID #44-59). These samples were integrated in compact sediment and the charred organic remains were first separated and placed in a sterilized petri dish with the help of a scalpel using a low power Leica M165C stereomicroscope in the *Laboratori d'Arqueologia* (*Universitat de València*). Selected remains were photographed using Leica Application Suite V3 and Helicon Focus 3.10.3 software.

The anatomical identification of the charred remains was carried out using a Leica DM6000 M optical microscope used in bright and dark field modes and polarization. Biometric measurements were recorded using Leica Applications Suite (LAS X). We used a Hitachi S-4100 and Hitachi S4800 scanning electron microscope (SEM) with spotlight of field emission and digital image acquisition system QUANTAX 200 to observe specific features and to obtain high-quality images. The microscope was operated at 10 kV and a 15-mm working distance. For SEM observation, the archaeological material was fixed by carbon tape to the slide, metallized with gold-palladium to facilitate

conductivity and subjected to vacuum. The elemental analyses were performed using a Hitachi S4800 SEM with a Brucker 1110 CHNS X-ray spectroscopy device and the ESPRIT 1.9 software.

Wood consists of organic matter: cellulose (~50%), lignin (20–30%), hemicellulose (15–25%) and a low proportion of minerals (~1%)[19]. The chemical elements in wood are carbon, oxygen, hydrogen and nitrogen; the latter and others occur in very low quantities. The proportion of these elements varies slightly from one species to another and even among the different parts of an individual plant[20]. According to Sawerysyn[19], the elemental composition of dry wood is 50% carbon, 44% oxygen and 6% hydrogen. Wood also contains small quantities of minerals, such as copper, magnesium and zinc, which are always below 1% of its mass, but they can be 10 times more abundant in the bark[20].

Elemental analyses using energy dispersive X-ray were carried out on two plant samples (samples ID #48-49) and compared with the results from other Palaeolithic sites to confirm burning. All measurements were made at 5000X.

2.6. Micromorphology

Micromorphology analysis was conducted on four thin sections, measuring 13.5 × 5.5 cm and with a thickness of 25 μm, prepared from three undisturbed sediment blocks: samples ID #60-62 (Supplementary Fig. S4), collected during the 2015 fieldwork campaign. The blocks were impregnated with a Palatal® P 4, an unsaturated polyester resin based on orthophthalic acid and standard glycols, dissolved in styrene. The four thin sections were manufactured by the micromorphology laboratory service of the *Departament de Medi Ambient i Ciències del Sòl* (*Universitat de Lleida*). Microscopic analysis was performed using a polarizing microscope (Nikon E600) at ×20, ×40, ×100, ×200 and ×400 magnifications under plane-polarized light (PPL), crossed polarized light (XPL), and oblique incident light (OIL). Microphotographs were taken using a Nikon Digital Sight DS-Fi2 camera. Micromorphological descriptions were made in line with standard guidelines[21–24]. The microfacies described were defined by groundmass, microstructure, organic and inorganic composition, and pedofeatures of the lithological changes within an individual thin section [25].

2.7. Organic chemistry

Lipid extraction, identification and quantification were carried out in the Archaeological Micromorphology and Biomarkers (AMBI) Lab of the *Instituto Universitario de Bio-Orgánica Antonio González* (*Universidad de La Laguna*). Two grams for each of two sediment samples taken from layers Xc and XI (samples ID #64 and #65, respectively) were dried at 60 °C for 48 h, then ground and homogenized. Non-volumetric material (including glassware and glass wool) was calcined at 450 °C for 10 h before extraction to eliminate any possible organic compounds. Lipids were extracted in 20 mL of 9:1 dichloromethane/methanol following a modified version of a previously published methodology [26]. Three 30-min sonicator and 10-min centrifuge (4700rpm) cycles were applied to the mixture. The total extract was then evaporated and separated using solid phase extraction (SPE) via silica gel column chromatography into six fractions of different polarities. In this paper, we present results for the *n*-alkane and aromatic fraction, eluted with $^{3}/_{8}$ of dead volume in *n*-hexane. After addition of 8 mg/L of 5α-androstane as internal standard (IS), the volume was completed with 150 µL of dichloromethane.

The presence of polycyclic aromatic hydrocarbons (PAHs) allows the identification of organic components of combustion structures, even under low combustion temperature conditions, as these molecular compounds are formed under conditions of thermal alteration[27,28]. Alkanes, the hydrocarbon molecules derived largely from epicuticular waxes of plants[29,30], are present in soils and sediments and are relatively resistant to biodegradation due to their relative hydrophobicity and indigestibility[31]. The distribution of C-numbers in *n*-alkanes within soil and sediment deposits helps identify their source material with terrestrial higher plants producing long-chain *n*-alkane ratios (carbon number C_{27} to C_{35}), while aquatic plants exhibit short- and medium-chain *n*-alkane ratios (C_{17} to C_{25}). The compounds in the *n*-alkane and aromatic fraction (polycyclic aromatic hydrocarbons or PAHs) were identified and quantified by gas chromatography using a coupled detection and mass-selective detector (GC-Agilent 7890B, MSD Agilent 5977A) equipped with an HP-5MS capillary column (30 m, ID: 250 µm, film thickness: 0.25 µm). The GC was programmed to an initial temperature of 70 °C for 2 min, heated with a heating rate of 12 °C/min to 140 °C and to final temperature of 320 °C with a heating rate of 3 °C/min and held for 15 min, with helium as the carrier gas (2 mL/min). To avoid overlap or interferences with other compounds, the total run time was 82.83 min. The multimode injector was held at a split ratio of 5:1 at an initial temperature of 70 °C for

0.85 min and heated to 300 °C at 720 °C/min. All measurements were repeated twice. The MS was operated in full scan mode (*m*/*z* 40–580) with an electron ionization energy of 70 eV and with the temperatures of the ion source and quadrupole set at 230 °C and 150 °C, respectively. Quantification was performed by taking the four most intense fragment ions (*m*/*z* 43, 57, 71 and 85, and *m*/*z* 67, 95, 81 and 245 for IS) for *n*-alkanes and the total ion chromatogram for the rest of the analyses. Compounds were identified by comparing their retention times and mass spectra with those of reference compounds (mix C_8-C_{40} alkanes and 5α-androstane, Supelco) and with the NIST mass spectra library. Quantification was based on calibration curves obtained by plotting the ratio Area/$Area_{IS}$ *vs* the concentration of each reference compound. Correlation coefficients were higher than 0.995. The *n*-alkane concentration is expressed as µg of individual compound per gram of dry sample (µg gds^{-1}).

2.8. Magnetic properties

In order to determine if the materials had been affected by past processes of heating, the magnetic properties of several archaeological samples were analysed (Supplementary Table S5). Eight lithic items in the assemblage present a colour that could be the result of thermal alteration[32] (Figure 7). Two of these samples underwent further analysis: ID #104 and #105. Both are quartzite pebbles, sample ID #104 presenting an intense reddish colour at one of its ends (Figure 7a). We also analysed two sediment samples (ID #93 and #94) located close to the potentially thermally altered zone as well as six unheated sediment control samples from the same stratigraphic unit (ID #95, #96, and #98-#100) located some distance from this zone but presenting the same lithology.

Additionally, cobbles representing three types of local quartzite (samples ID #101-#103) similar to that found at the site were heated under controlled temperature conditions so as to analyse any variation in their respective magnetic properties (Supplementary Figure S7). Fragments of these pebbles were first tested at room temperature and then heated to 300 ºC and 600 ºC in a TD48-SC (ASC) magnetically shielded oven for 1 h, their magnetic properties being measured after each heating step. The bulk sample (~450 mg) of both the archaeological and experimental materials was measured using a variable field translation balance. The analyses included progressive isothermal remanent magnetization (IRM) acquisition curves, hysteresis cycles (±1T), backfield coercivity

curves and thermomagnetic curves up to 700 ºC in air. These analyses were carried out at Burgos University's (Spain) palaeomagnetism laboratory.

The success of rock-magnetic methods for identifying heating processes in archaeological materials largely depends on the detection of significant differences in their magnetic properties – that is, on the composition, concentration and granulometry of their ferromagnetic minerals. The formation of magnetite and/or maghaemite is probably the most common transformation process underwent by the iron-bearing minerals present in heated archaeological materials. These minerals are hundreds of times more magnetic than others, including, for example, haematite [33], which means that differences in the intensity of the magnetic signal are a useful criterion for identifying heating. However, the neoformation of magnetite is not always recorded[34] and the magnetic properties exhibited by the material will depend on the conditions under which heating took place and the ferromagnetic minerals that were created.

SUPPLEMENTARY TEXT 3

3. Other organic materials

3.1. Bone remains

Samples ID #45 and #47 were brown bones with no thermal alteration while samples ID #44, #46, #48, #51-#52, #55 and #58 were dark, probably burnt bones. Microscopic analysis showed all samples to present the cellular structure of compact bone tissue with osteons formed by concentric layers around the Havers channels. The osteons are separated by cement lines. The bone was highly altered with a friable consistency. At the lowest pressure, the bone was found to disintegrate.

3.2. Humus

Samples ID #50-#54, #56-#57 and #59 can be classified as humus. The general appearance of these samples was dark, unstructured organic matter. We classified them as humus because no clear remains of plant or animal cells could be identified. Only in sample ID #51 did we identify some small particles of plant, bone and calcium, the latter possibly representing decaying bones.

Supplementary Figure S1. a. Detailed view of the Almonda escarpment showing the location of Gruta da Aroeira. **b.** Stratigraphic profile showing the position of the Aroeira 3 cranium (red star) and the main horizon with burnt elements.

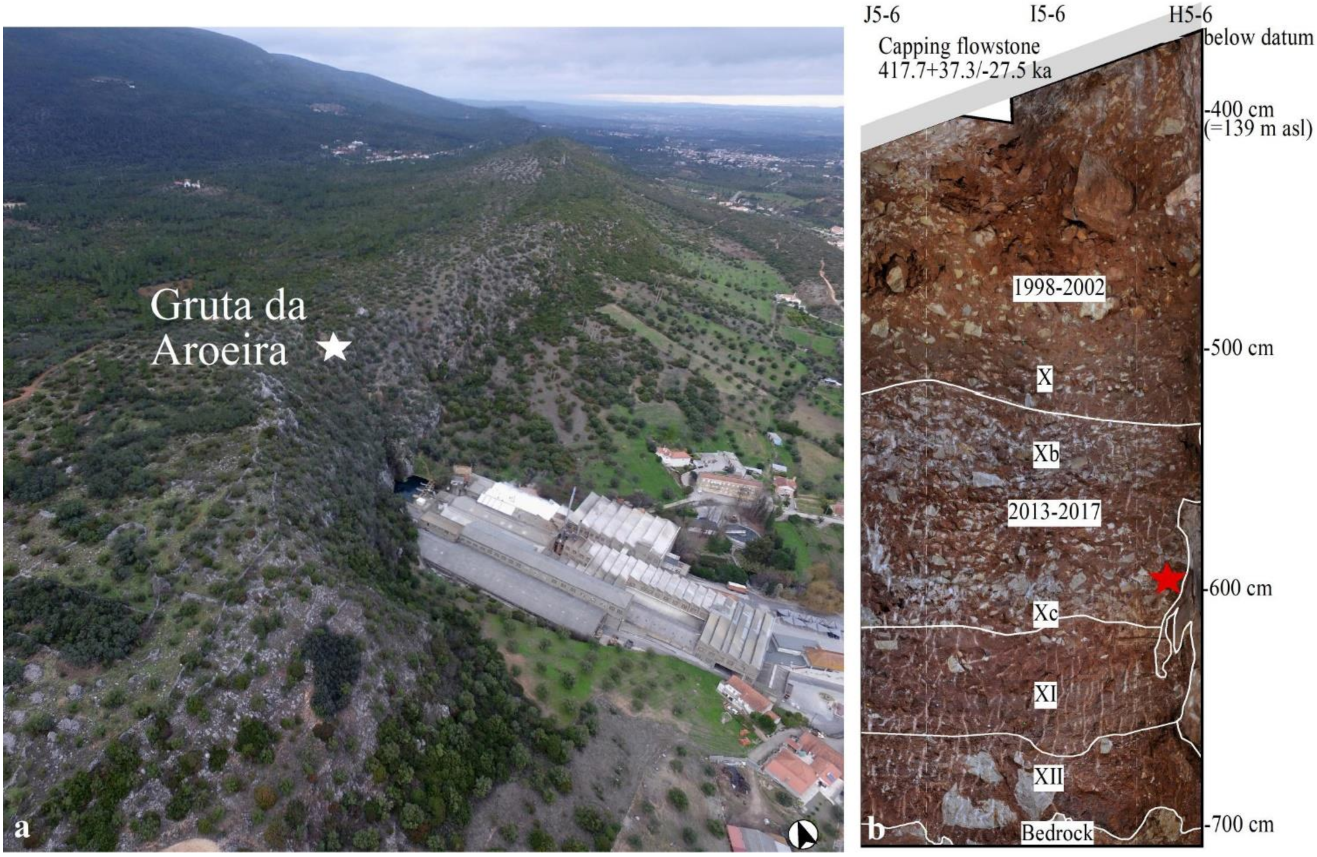

Supplementary Figure S2. Control samples. **a.** Manganese coating on deer tooth (sample ID #90). **b.** Manganese coating on long bone (sample ID #91). **c.** Unburnt bone with no manganese coating (sample ID #92).

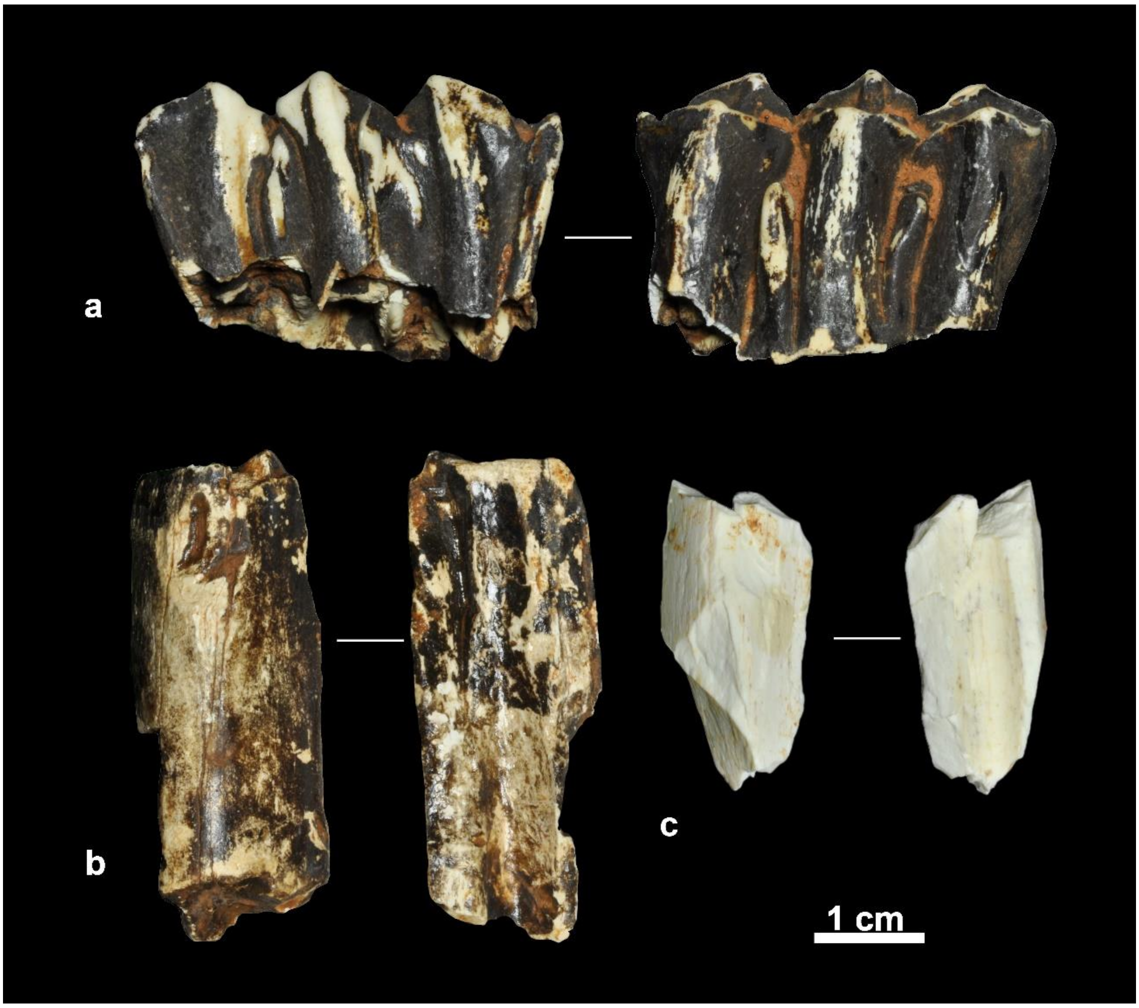

Supplementary Figure S3. Elemental proportions in plant tissue samples from Aroeira.

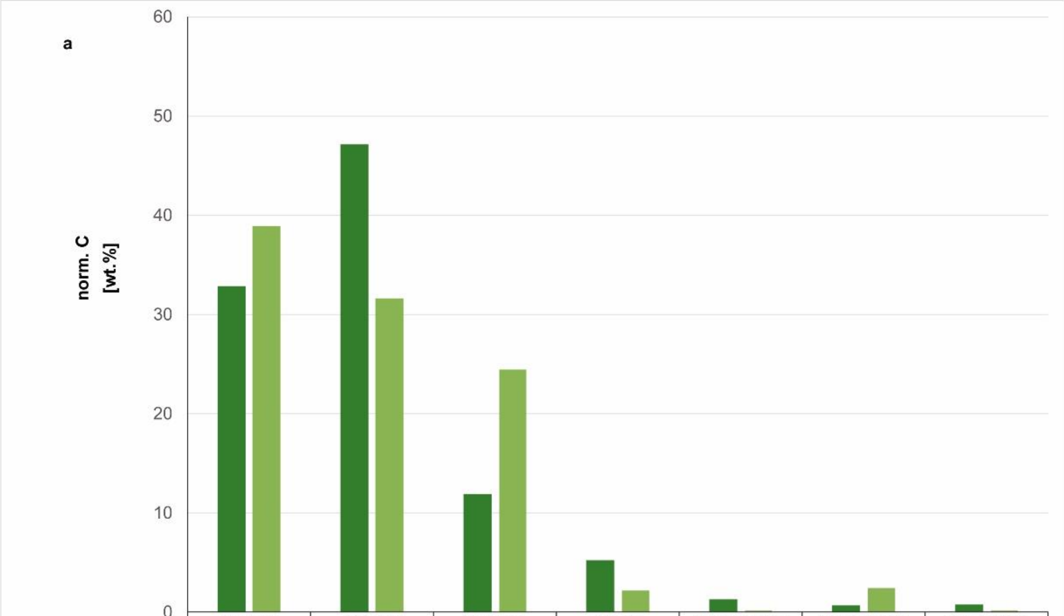

	Carbon	Oxygen	Calcium	Silicon	Magnesium	Phosphorus	Potassium
Sample ID #48	32.88	47.20	11.88	5.27	1.30	0.66	0.82
Sample ID #49	38.94	31.67	24.41	2.21	0.18	2.42	0.17

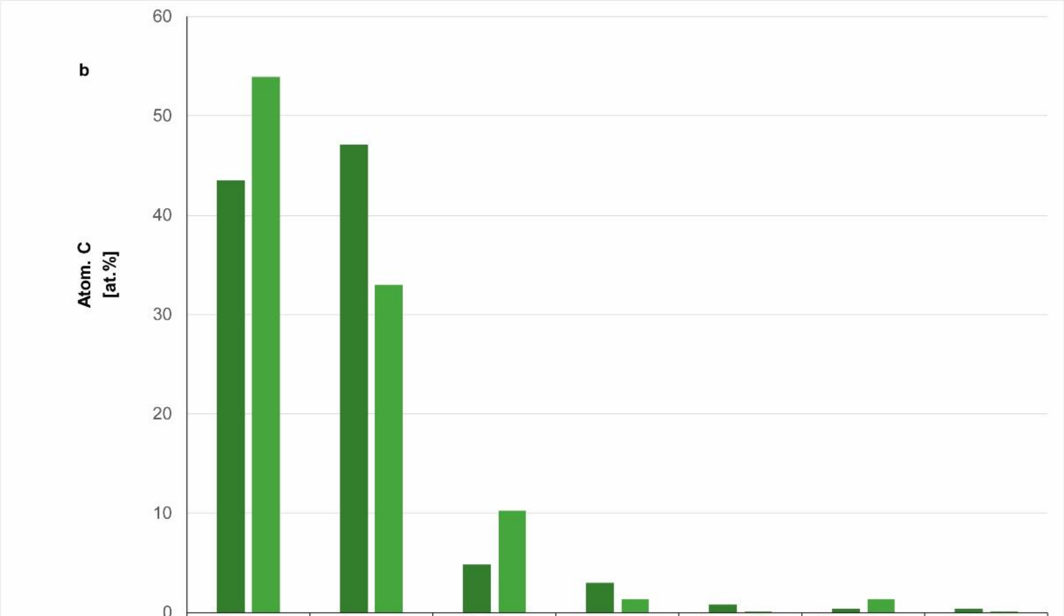

	Carbon	Oxygen	Calcium	Silicon	Magnesium	Phosphorus	Potassium
Sample ID #48	43.47	47.13	4.87	3.01	0.84	0.35	0.33
Sample ID #49	53.88	32.98	10.30	1.33	0.12	1.32	0.07

Supplementary Figure S4. a. The sequence of four thin-section scans made from the three blocks removed from the column shown in "b" (samples ID #60-#62). **b.** General view of the stratigraphic profile and the three micromorphological samples (indicated in yellow rectangles) spanning the unit where most burnt bones were recovered.

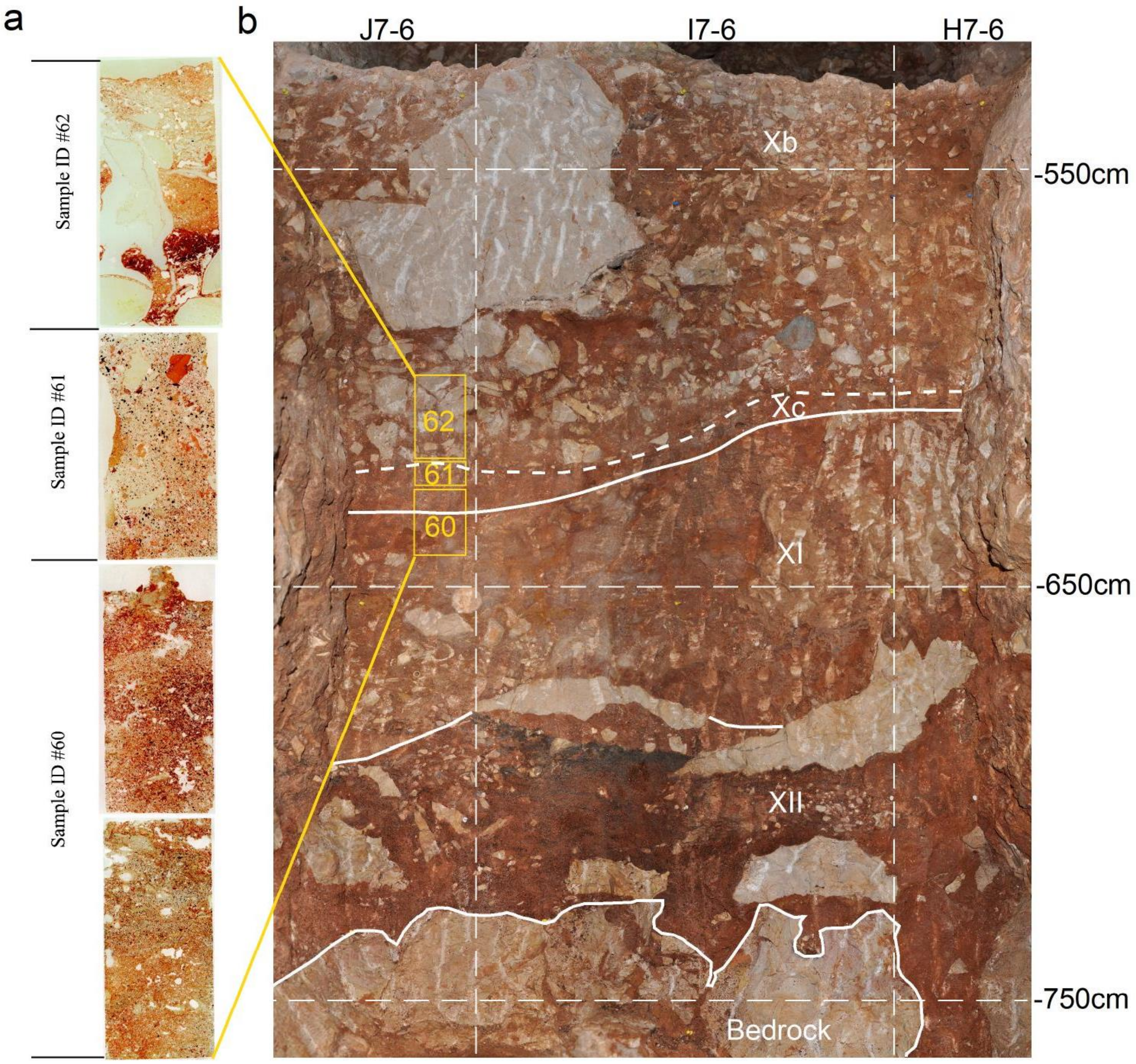

Supplementary Figure S5. *N*-Alkane barplot from samples ID #63 and #64 (layers Xc and XI, respectively).

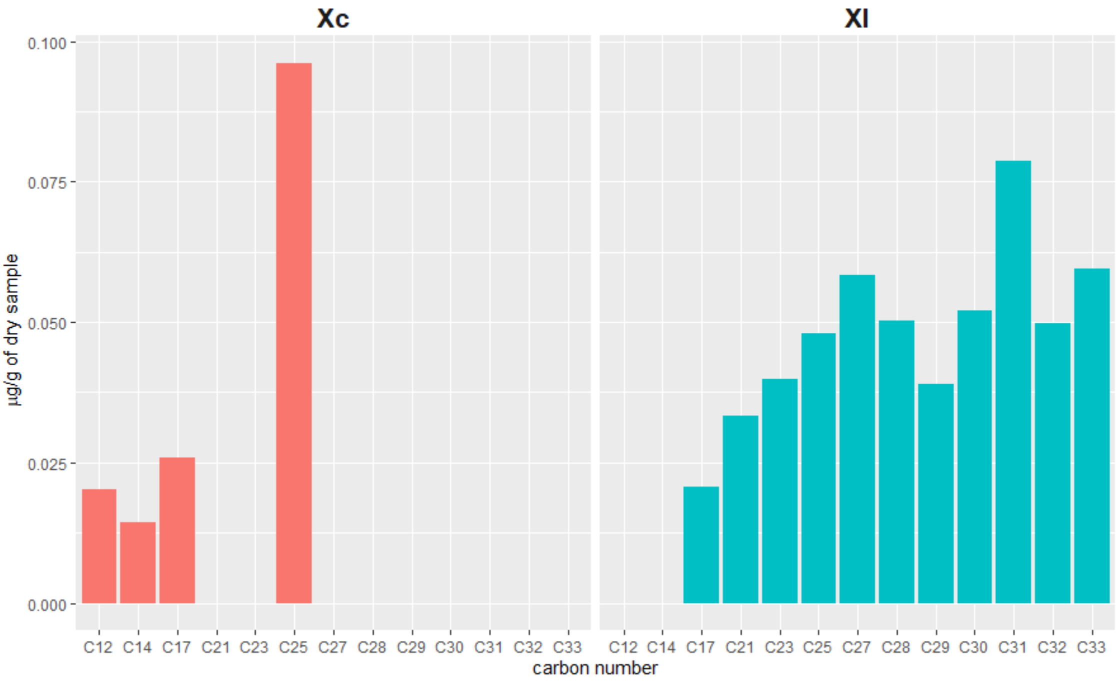

Supplementary Figure S6. Thermomagnetic curves (magnetization vs. temperature) of (**a**) representative sample of sediment lying close to the possible thermally altered area (sample ID #93) and (**b**) representative sample of sediment lying some distance from the possible thermally altered area (sample ID #99). Heating (cooling) cycles are shown in red (blue) with their respective arrows. Sample code and magnetization values are also indicated.

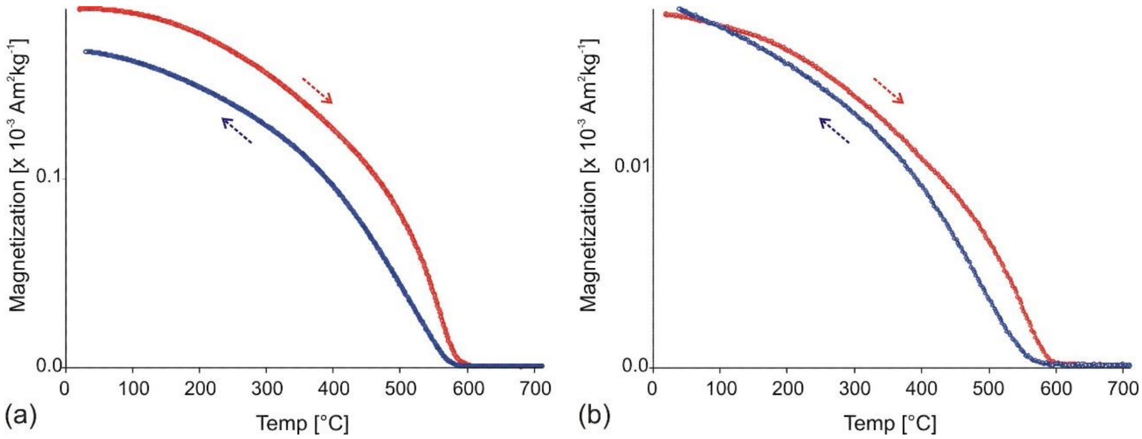

Supplementary Figure S7. Local quartzite control samples ID: #101 (**a**), #102 (**b**) and #103 (**c**).

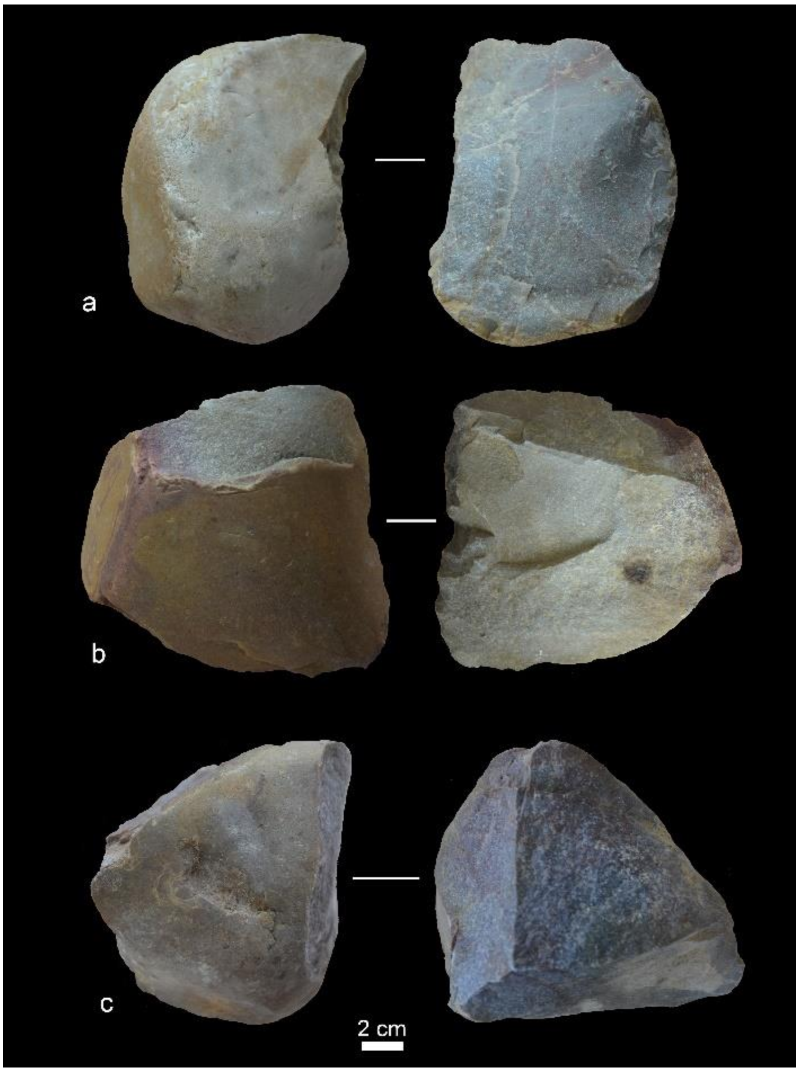

Supplementary Figure S8. Variation in the intensity of magnetization as a function of temperature for three local quartzites (samples ID# 101-103). Each point represents the magnetization value in the heating cycle of the thermomagnetic curve at 30 ºC (J_{30}) after three different increases in temperature: room temperature, 300 ºC and 600 ºC. Symbols for samples ID #104-105 are the two archaeological quartzites measured at room temperature. Note the high value of sample ID #104_2d in comparison with its counterparts.

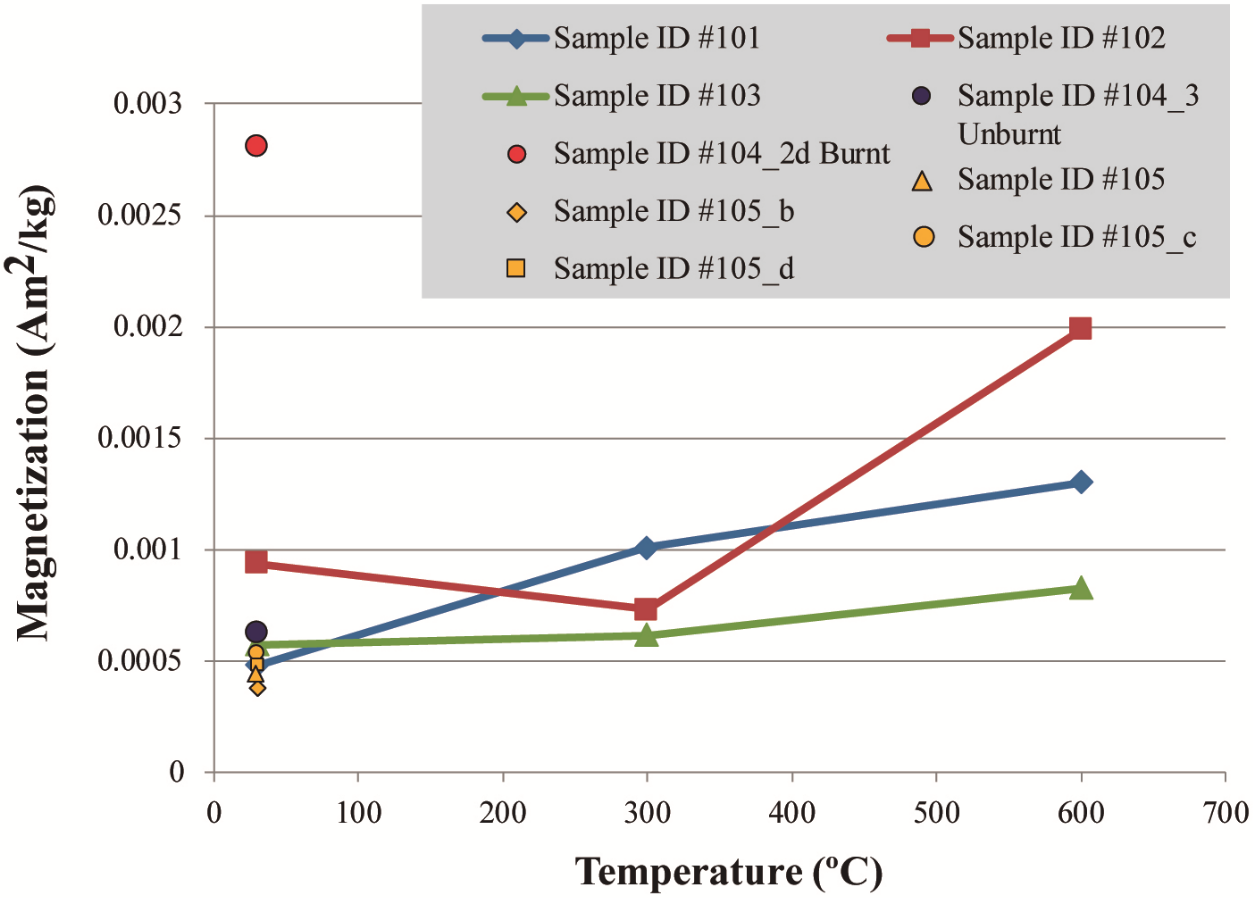

Supplementary Table S1. List of all the samples discussed in this paper (taphonomic and zooarchaeological analyses of bones, wood charcoal, micromorphology, organic chemistry, FTIR, XEDS, and magnetic properties analyses).

Sample ID	Site #	Layer	Material	Analysis	Square	Description
**1**	124-1	Xc	Burnt bone	Taphonomy/zooarchaeology/FTIR	J7	Long bone
**2**	124-2	Xc	Burnt bone	Taphonomy/zooarchaeology	J7	Flat bone
**3**	130	Xc	Burnt bone	Taphonomy/zooarchaeology/FTIR/XEDS	H7	Metapodial
**4**	132	Xc	Burnt bone	Taphonomy/zooarchaeology	I7	Epiphysis frag
**5**	140	XI	Burnt bone	Taphonomy/zooarchaeology	J7	Long bone
**6**	343	Xb	Burnt bone	Taphonomy/zooarchaeology/FTIR/XEDS	I6	Bone scute
**7**	408	Xb	Burnt bone	Taphonomy/zooarchaeology	H6	Epiphysis
**8**	559	Xc	Burnt bone	Taphonomy/zooarchaeology	J6	Flat bone
**9**	568	Xc	Burnt bone	Taphonomy/zooarchaeology	J6	Long bone
**10**	586	Xc	Burnt bone	Taphonomy/zooarchaeology/FTIR	I6	Long bone
**11**	587	Xb/c	Burnt bone	Taphonomy/zooarchaeology/FTIR	I6	Flat bone
**12**	607	Xb	Burnt bone	Taphonomy/zooarchaeology	H6	Long bone
**13**	610	Xc	Burnt bone	Taphonomy/zooarchaeology/FTIR	I6	Rib
**14**	615	Xc	Burnt bone	Taphonomy/zooarchaeology/FTIR	I6	Spongy fragment
**15**	618	Xc	Burnt bone	Taphonomy/zooarchaeology/FTIR	I6	Flat bone
**16**	621	Xc	Burnt bone	Taphonomy/zooarchaeology/FTIR/XEDS	I6	Flat bone
**17**	622	Xc	Burnt bone	Taphonomy/zooarchaeology	I6	Spongy fragment
**18**	623	Xc	Burnt bone	Taphonomy/zooarchaeology/FTIR/XEDS	I6	Mandible
**19**	628	Xc	Burnt bone	Taphonomy/zooarchaeology	I6	Long bone
**20**	629	Xc	Burnt bone	Taphonomy/zooarchaeology/FTIR/XEDS	I6	Spongy fragment
**21**	631	Xc	Burnt bone	Taphonomy/zooarchaeology	I6	Flat bone
**22**	632	Xc	Burnt bone	Taphonomy/zooarchaeology	I6	Spongy fragment
**23**	635	Xc	Burnt bone	Taphonomy/zooarchaeology	I6	Spongy fragment
**24**	637	Xc	Burnt bone	Taphonomy/zooarchaeology/FTIR/XEDS	J6	Flat bone
**25**	641	Xc	Burnt bone	Taphonomy/zooarchaeology	I6	Spongy fragment
**26**	642	Xc	Burnt bone	Taphonomy/zooarchaeology	I6	Root
**27**	645	Xc	Burnt bone	Taphonomy/zooarchaeology	I6	Rib
**28**	729	Xc	Burnt bone	Taphonomy/zooarchaeology	I6	Spongy fragment
**29**	732	Xb	Burnt bone	Taphonomy/zooarchaeology	I5	Vertebra
**30**	881	Xb	Burnt bone	Taphonomy/zooarchaeology/FTIR/XEDS	I8	Vertebra
**31**	1438	Xc/XI	Burnt bone	Taphonomy/zooarchaeology	J7	Flat bone
**32**	1440	Xc/XI	Burnt bone	Taphonomy/zooarchaeology	J7	Flat bone
**33**	1441	Xc/XI	Burnt bone	Taphonomy/zooarchaeology/FTIR	J7	Flat bone
**34**	1452	Xc/XI	Burnt bone	Taphonomy/zooarchaeology	J7	Spongy fragment
**35**	1454	Xc/XI	Burnt bone	Taphonomy/zooarchaeology	J7	Spongy fragment
**36**	1459	Xc/XI	Burnt bone	Taphonomy/zooarchaeology	J7	Long bone
**37**	1466	Xc/XI	Burnt bone	Taphonomy/zooarchaeology	J7	Spongy fragment
**38**	1471	Xc/XI	Burnt bone	Taphonomy/zooarchaeology	J7	Epiphysis frag
**39**	1472	Xc/XI	Burnt bone	Taphonomy/zooarchaeology	J7	Spongy fragment
**40**	1488	Xc/XI	Burnt bone	Taphonomy/zooarchaeology	J7	Epiphysis frag
**41**	1498	Xc/XI	Burnt bone	Taphonomy/zooarchaeology/FTIR	J7	Flat bone
**42**	1502	Xc/XI	Burnt bone	Taphonomy/zooarchaeology	J6	Flat bone
**43**	1503	Xc/XI	Burnt bone	Taphonomy/zooarchaeology	J7	Spongy fragment
**44**	565	Xc	Bone	Wood charcoal analysis	J6	Probably burnt
**45**	650	Xc	Bone	Wood charcoal analysis	J6	
**46**	566	Xc	Bone	Wood charcoal analysis	J6	Probably burnt
**47**	620	Xc	Bone	Wood charcoal analysis	I6	
**48**	644	Xc	Charcoal and bone	Wood charcoal analysis	I6	Probably burnt bone
**49**	1443	Xc/XI	Charcoal	Wood charcoal analysis	J7	
**50**	1444	Xc/XI	Humus	Wood charcoal analysis	J7	
**51**	1457	Xc/XI	Charcoal and bone	Wood charcoal analysis	J7	Probably burnt bone
**52**	1458	Xc/XI	Bone and humus	Wood charcoal analysis	J7	Probably burnt bone
**53**	1460	Xc/XI	Humus	Wood charcoal analysis	J7	
**54**	1463	Xc/XI	Humus and microorganism	Wood charcoal analysis	J7	

Sample ID	Site #	Layer	Material	Analysis	Square	Description
**55**	1469	Xc/XI	Bone	Wood charcoal analysis	J7	Probably burnt
**56**	1480	Xc/XI	Humus	Wood charcoal analysis	J7	
**57**	1497	Xc/XI	Bone and humus	Wood charcoal analysis	J7	
**58**	1499	Xc/XI	Bone and humus	Wood charcoal analysis	J7	Probably burnt bone
**59**	1504	Xc/XI	Humus	Wood charcoal analysis	J7	
**60**	560	Xc/XI	Sediment	Micromorphology	H6	Thin section
**61**	556	Xc	Sediment	Micromorphology	J6	Thin section
**62**	522	Xb	Sediment	Micromorphology	I6	Thin section
**63**	–	Xc	Sediment	Organic chemistry	–	
**64**	–	XI	Sediment	Organic chemistry	–	
**65**	580	Xb	Unburnt bone	FTIR	I6	
**66**	254	Xb	Unburnt bone	FTIR	I6	
**67**	277	Xb	Unburnt bone	FTIR	I6	
**68**	880	Xb	Unburnt bone	FTIR	J8	
**69**	543	Xb	Unburnt bone	FTIR	I6	
**70**	831	Xb	Unburnt bone	FTIR	I8	
**71**	314	Xb	Unburnt bone	FTIR	I6	
**72**	235	Xb	Unburnt bone	FTIR	J6	
**73**	251	Xb	Unburnt bone	FTIR	I6	
**74**	386	Xb	Unburnt bone	FTIR	I6	
**75**	456	Xb	Unburnt bone	FTIR	I6	
**76**	928	XI	Unburnt bone	FTIR	I7	
**77**	96	Xb	Sediment	FTIR	J7	Red clay. Not rubefacted sediment
**78**	19	F	Sediment	FTIR	H7	Not rubefacted sediment
**79**	20	F	Sediment	FTIR	I7	Not rubefacted sediment
**80**	586-1	X	Sediment	FTIR	I6	Sediment attached to burnt bone #586
**81**	551	Xb	Sediment	FTIR	I6	Not rubefacted sediment
**82**	54	Xb	Sediment	FTIR	I7	Not rubefacted sediment
**83**	918	Xb	Sediment	FTIR	I6	Not rubefacted sediment
**84**	928-1	XI	Sediment	FTIR	I7	Sediment attached to unburnt bone #928
**85**	615-1	Xc	Sediment	FTIR	I6	Sediment attached to burnt bone #615
**86**	1498-1	Xc	Sediment	FTIR	J7	Sediment attached to burnt bone #1498-1
**87**	1508	Xc	Sediment	FTIR	J7	
**88**	572	Xc	Sediment	FTIR	J6	Reddish sediment
**89**	–	XI	Sediment	FTIR	H7	Black nodule
**90**	Praia 1	–	Deer tooth	XEDS	–	Manganese coating. Control sample
**91**	Praia 2	–	Bone	XEDS	–	Manganese coating. Control sample
**92**	332	Xb	Bone	XEDS	I6	Unburnt bone. Control sample
**93**	1444	Xc	Sediment	Magnetic properties	J7	Sediment with burnt bones
**94**	1442	Xc	Sediment	Magnetic properties	J7	Sediment with burnt bones
**95**	738	XI	Sediment	Magnetic properties	I5	Sediment attached to quartzite #738. Unheated sediment.
**96**	156	XI	Sediment	Magnetic properties	I7	Sediment attached to unburnt bone #156
**97**	238	Xb	Sediment	Magnetic properties	J6	Sediment attached to unburnt bone #238
**98**	2017-1	Xa	Sediment	Magnetic properties	J6-7-8	Unheated sediment. Control sample
**99**	898	Xb	Sediment	Magnetic properties	H6	Sediment attached to unburnt bone #898
**100**	2014-1	Xb	Sediment	Magnetic properties	I-J-H6	Unheated sediment. Control sample
**101**	Quartzite 1	–	Experimental quartzite cobble	Magnetic properties	–	Local quartzite from Aroeira surroundings. Experimental analysis

Sample ID	Site #	Layer	Material	Analysis	Square	Description
**102**	Quartzite 2	–	Experimental quartzite cobble	Magnetic properties	–	Local quartzite from Aroeira surroundings. Experimental analysis
**103**	Quartzite 3	–	Experimental quartzite cobble	Magnetic properties	–	Local quartzite from Aroeira surroundings. Experimental analysis
**104**	262	Xb	Archaeological quartzite cobble	Magnetic properties	I6	Possible thermal alteration
**105**	591	Xb	Archaeological quartzite cobble	Magnetic properties	I6	Possible thermal alteration
**106**	265	Xb	Archaeological quartzite cobble		I6	Possible thermal alteration
**107**	334	Xb	Archaeological quartzite cobble		I6	Possible thermal alteration
**108**	846	Xb	Archaeological quartzite cobble		I8	Possible thermal alteration
**109**	353	Xb	Archaeological quartzite cobble		I6	Possible thermal alteration
**110**	299	Xb	Archaeological quartzite cobble		I6	Possible thermal alteration
**111**	340	Xb	Archaeological quartzite cobble		I6	Possible thermal alteration

Supplementary Table S2. Degree of burning presented by thermally modified bones from layer X based on visual alteration[12].

Burning degree	Description	NR	%NR
1	slightly burned	2	5
2	more than half carbonized	7	16
3	fully carbonized	22	51
4	slightly calcined	2	5
5	more than half calcined	6	14
6	fully calcined with the colour completely white	4	9
**Total**		**43**	**100**

Supplementary Table S3. Energy-dispersive X-ray spectroscopy results for the samples analysed.

Sample ID	Site #	C	N	O	F	Na	Mg	Al	Si	P	S	Cl	K	Ca	Ti	Mn	Fe	Cu	Zn
3	130	34.76		22.54						8				32.82				0.83	1.05
3	130	21.8		32.53					0.15	0.44				43.9				1.19	
3	130	31.2		55.95		0.34				5.04				7.22				0.13	0.12
6	343	13.69		66		0.22				8.41				11.53				0.15	
6	343	23.25		63.08		0.18		0.15	0.18	5.43				7.56				0.17	
6	343	14.08		63.68		0.18		1.86	2.13	6.99			0.06	10.09			0.45	0.22	0.27
16	621	16.7		58.58	1.11	0.38				9.12				13.85				0.26	
16	621	20.37		53.37	0.84	0.38				9.32		0.13		15.04				0.24	0.3
16	621	18.02		64.79		0.13	0.2	5.95	5.6	1.09			0.14	1.57	0.09		2.28	0.14	
16	621	24.4		65.41				1.3	1.19					7.39			0.17	0.15	
18	623	70.3		24.49			2.29	0.57	1.7								0.37	0.14	0.14
18	623	47.16	8.93	37.26		0.24	2.62		3.09		0.18	0.14	0.14	0.04				0.1	0.1
18	623	11.24		63.48			11.06		14.01									0.21	
20	629	38.61		52.29				0.15	0.14	1.27				7.18			0.08	0.16	0.12
20	629	25.11		51.23		0.18		0.18	0.14	4.68				17.68				0.46	0.33
20	629	27.74		43.37				0.16	0.17	0.37				26.96				0.62	0.6
24	637	17.38		53.11	1	0.4		0.61	0.38	10.31		0.12		16.4				0.3	
24	637	22.12		59.98	1.57	0.31		0.15	0.16	5.08				10.32				0.14	0.16
30	881	22.59		62.19										15.22					
30	881	24.3		59.36				0.38	0.42	0.16				14.95			0.22	0.21	
30	881	36.64		21.71						3.42				36.4				1.82	
30	881	41.36		49.94		0.24				3.59				4.87					
90	Praia 1	50.99		38.41			0.1	2.88	1.44	0.15				1.13	0.08	3.02	1.58	0.21	
91	Praia 2	34.4		53.78			0.16	2.14	1.45	2.08			0.07	3.29		1.68	0.7	0.11	0.14
91	Praia 2	30.88		56.38			0.22	3.68	2.31	0.65			0.07	1.41		3.04	1.07	0.16	0.16
92	332	15.93		59.8						9.38				14.6				0.29	
92	332	12.38		68.78		0.28				7.52				10.64				0.21	0.18
92	332	13.13		67.34		0.25				7.8				11.04				0.25	0.19

Supplementary Table S4. a. Superposition of four thin-section scans made from the three blocks removed from the column shown in "Supplementary Figure S4" with the microfacies identified. **b.** Description of the 12 microfacies identified.

a

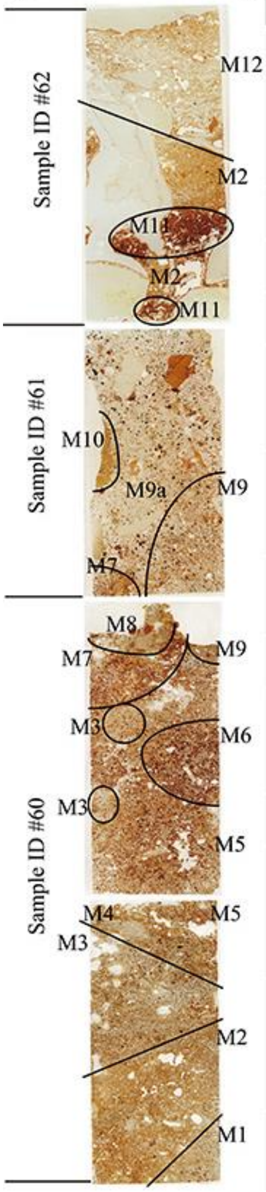

b

Microfacies	Layer	Samples ID#	Characteristics
**M12**	Xb	62	Crumbly clay matrix and very fine material. Ageing of earthworm excrements. The voids are deformed and strongly coalesced with presence of thin dark brown coatings of fine material probably due to water percolation. Infilled voids characterized by sparitic calcite. No bone fragments.
**M11**	Xb	62	Thick monomorphic clay coating with polygonal cracking. Sharp contact with M12.
**M10**	Xc	61	Granular clay infillings with subangular quartz fragments (mm-sized).
**M9a**	Xc	61	Same as 9 but with cm-sized bone fragments, diagenetically altered.
**M9**	Xc	61<br>60	Massive heterogeneous fine matrix (silty clay) with lenticular microstructure. Small voids and channels due to bioturbation. Presence of abundant clay nodules and few charcoal flecks.
**M8**	Xc	60	Fossilized iron-stained coarse subangular fragments (cm-sized) with sparitic calcite infillings. Some phosphate nodules probably related to coprolite fragments. Few charcoal flecks.
**M7**	Xc	61<br>60	Granular clay matrix with lenticular microstructure, brownish-red and partially weathered fragments of clay coatings within the matrix. Some scattered sparitic calcite.
**M6**	Xc	60	As 3 and 5 but with massive granular clay matrix and Fe-Mn rich nodules.
**M5**	Xc	60	Similar to 3 but with less sparitic calcite presence. Deformed infilled voids and channels from mesofaunal activity. Abundant sub-rounded bone fragments (cm-sized the larger), some thermoaltered.
**M4**	Xc	60	Massive cemented sparitic calcite with charcoal dust or flecks.
**M3**	Xc	60	Fine clay lamination with Fe-rich nodules and sparitic limestone fragments together with light gray sparitic calcite. Charcoal flecks. Thermoaltered sub-rounded bone fragments (cm-sized), voids, and channels from mesofaunal activity.
**M2**	XI<br>Xb	60	Granular clay lamination with clay nodules and hypocoatings. Fine graded bedding with presence of secondary sparitic calcite. No bone fragments. Modified planar and complex voids related to mesofauna bioturbation.
**M1**	XI	60	Granular clay lamination with clay nodules and hypocoatings. Fine graded bedding with presence of secondary sparitic calcite. No bone fragments.

Supplementary Table S5. Magnetic properties of materials studied, including archaeological and experimental quartzites and archaeological sediment samples. From left to right: J_{30} (magnetization value of the heating cycle of the thermomagnetic curve at 30 ºC); Ms (Saturation magnetization), Mrs (remanent saturation magnetization), Bc (coercive field) and Bcr (remanent coercive field). "Non interp." = non-interpretable thermomagnetic curves. Units for J_{30}, Ms and Mrs are Am^2kg^{-1}. Units for Bc and Bcr are milliteslas or mT. In the experimental quartzites the code "PRE" refers to samples measured at room temperature (unheated) and "300" and "600" refer to samples heated to 300 ºC and 600 ºC, respectively.

Material	Sample ID#	Sample code	J_{30}	Ms	Mrs	Bc	Bcr	Observations
Archaeological quartzite cobbles	104	ARO262_2 unburnt	*Non interp.*	6.63E-04	2.06E-04	27.5	109	cobble #262
	104	ARO262_3 unburnt	6.31E-04	8.00E-04	2.33E-04	26.5	111.6	cobble #262
	104	ARO_262_2a-burnt	*Non interp.*	1.21E-03	2.86E-04	18.2	29.3	cobble #262 (reddened end)
	104	ARO_262_2d-burnt	2.81E-03	7.54E-03	8.81E-04	13.9	32.4	cobble #262 (reddened end)
	105	ARO_591	5.00E-04	8,55E-04	1.52E-04	11.7	30.1	cobble #591
	105	ARO_591b	4.10E-04	8.31E-04	1.56E-04	12	28	cobble #591
	105	ARO_591c	5.80E-04	7.65E-04	1.49E-04	11.8	32.4	cobble #591
	105	ARO_591d	4.80E-04	6.76E-04	1.15E-04	19.9	34.1	cobble #591
Experimental quartzites	101	1D_PREa	3.12E-04	3.14E-04	6.11E-05	14.8	65.1	Quartzite 1– room temp.
	101	1D_300a	4.15E-04	4.36E-04	8.86E-05	16.4	48.2	Quartzite 1– heated 300ºC
	101	1D_600a	8.50E-04	1.83E-03	5.73E-04	21	28	Quartzite 1– heated 600ºC
	102	2B_PREa	9.39E-04	2.56E-03	4.04E-04	22.8	291	Quartzite 2– room temp.
	102	2B_300a	7.30E-04	8.03E-04	3.79E-04	61.8	409.5	Quartzite 2– heated 300ºC
	102	2B_600a	1.99E-03	2.73E-03	1.08E-03	24.5	37.8	Quartzite 2– heated 600ºC
	103	3D_PREa	5.75E-04	4.27E-04	2.90E-04	114.4	436.5	Quartzite 3– room temp.
	103	3D_300a	6.18E-04	3.59E-04	3.16E-04	103.8	358.6	Quartzite 3– heated 300ºC
	103	3D_600a	8.27E-04	1.12E-03	4.23E-04	35	98.4	Quartzite 3– heated 600ºC
Archaeological sediment samples	93	ARO-17.1444	1.18E-01	3.04E-01	5.23E-02	8.1	17.6	Burnt sample
	94	ARO-17.1442	2.01E-01	3.56E-01	5.21E-02	7.8	17.1	Burnt sample
	97	NQ-Xb-J6-238	1.87E-02	3.63E-02	4.63E-03	7.4	17.8	Unburnt control sample
	100	NQ-Xb-I-J-H6	2.42E-02	3.56E-02	6.34E-03	7.1	16.9	Unburnt control sample
	99	NQ-H6-Xb-898	1.78E-02	2.72E-02	4.90E-03	7.7	17	Unburnt control sample
	96	NQ_XI-I7-156	1.15E-01	1.86E-01	2.82E-02	7.4	16.5	Unburnt control sample
	98	NQ-Xa-J6-7-8	3.56E-02	5.28E-02	9.62E-03	7.7	17.3	Unburnt control sample
	95	NQ_XI-IS-738	2.67E-01	4.20E-01	7.58E-02	8.5	17.9	Unburnt control sample

References:

1. Zilhão, J., Maurício, J. & Souto, P. Jazidas arqueológicas do sistema cársico da nascente do Almonda. *Nov. Augusta* **7**, 35–54 (1993).

2. Marks, A. E. *et al.* Le gisement Pléistocène moyen de Galeria Pesada, (Estrémadure, Portugal) : premiers résultats. *Paléo* **14**, 77–100 (2002).

3. Hoffmann, D. L., Pike, A. W. G., Wainer, K. & Zilhão, J. New U-series results for the speleogenesis and the Palaeolithic archaeology of the Almonda karstic system (Torres Novas, Portugal). *Quat. Int.* **294**, 168–182 (2013).

4. Daura, J. *et al.* New Middle Pleistocene hominin cranium from Gruta da Aroeira (Portugal). *Proc. Natl. Acad. Sci.* **114**, 3397–3402 (2017).

5. Croitor, R., Sanz, M. & Daura, J. Deer remains from the Middle Pleistocene site of Gruta da Aroeira (Portugal): Iberian faunal endemism and implications for hominin paleobiogeography. *Quat. Sci. Rev.* **225**, 106022 (2019).

6. Alba, D. M. *et al.* New macaque remains from the Middle Pleistocene of Gruta da Aroeira (Almonda karst system, Portugal). *J. Hum. Evol.* **131**, 40–47 (2019).

7. Marks, A. E. *et al.* Excavations at the late Middle Pleistocene cave of Galeria Pesada: 1997/1999. *O Arqueólogo Port.* **20**, 7–38 (2002).

8. Reitz, E. J. & Wing, E. S. Zooarchaeology. (2008).

9. Stiner, M. C. *The faunas of Hayonim Cave, Israel: A 200,000-year record of Paleolithic diet, demography, and society*. (Harvard University Press, USA, 2005).

10. Fernández-Jalvo, Y. & Andrews, P. *Atlas of Taphonomic Identifications*. (Springer Netherlands, 2016). doi:10.1007/978-94-017-7432-1

11. Lyman, R. L. *Vertebrate Taphonomy*. *Cambridge University Press* (Cambridge University Press, 1994). doi:10.1017/CBO9781139878302

12. Stiner, M. C., Kuhn, S. L., Weiner, S. & Bar-Yosef, O. Differential burning, recrystallization, and fragmentation of archaeological bone. *J. Archaeol. Sci.* **22**, 223–237 (1995).

13. Poduska, K. M. *et al.* Decoupling Local Disorder and Optical Effects in Infrared Spectra: Differentiating Between Calcites with Different Origins. *Adv. Mater.* **23**, 550–554 (2011).

14. Regev, L., Poduska, K. M., Addadi, L., Weiner, S. & Boaretto, E. Distinguishing between calcites formed by different mechanisms using infrared spectrometry: archaeological applications. *J. Archaeol. Sci.* **37**, 3022–3029 (2010).

15. Berna, F. *et al.* Sediments exposed to high temperatures: reconstructing pyrotechnological processes in Late Bronze and Iron Age Strata at Tel Dor (Israel). *J. Archaeol. Sci.* **34**, 358–373 (2007).

16. Weiner, S. & Bar-Yosef, O. States of preservation of bones from prehistoric sites in the Near East: A survey. *J. Archaeol. Sci.* **17**, 187–196 (1990).

17. Wright, L. E. & Schwarcz, H. P. Infrared and Isotopic Evidence for Diagenesis of Bone Apatite at Dos Pilas, Guatemala: Palaeodietary Implications. *J. Archaeol. Sci.* **23**, 933–944 (1996).

18. Surovell, T. A. & Stiner, M. C. Standardizing Infra-red Measures of Bone Mineral Crystallinity: an Experimental Approach. *J. Archaeol. Sci.* **28**, 633–642 (2001).

19. Sawerysyn, J. La combustion du bois et ses impacts sur la qualité de l'air. *Air Pur* (2012).

20. Ragland, K. W., Aerts, D. J. & Baker, A. J. Properties of wood for combustion analysis. *Bioresour. Technol.* **37**, 161-168 (1991). doi:10.1016/0960-8524(91)90205-X

21. Bullock, P., Fedoroff, N., Jongerius, A., Stoops, G. & Tursina, T. *Handbook for soil thin section description*. (Waine Research, Wolverhampton, UK., 1985).

22. Courty, M.-A., Goldberg, P. & Macphail, R. I. Soils and micromorphology in archaeology. (Cambridge University Press, Cambridge, UK, 1989).

23. Stoops, G., Marcelino, V. & Mees, F. *Interpretation of Micromorphological Features of Soils and Regoliths*. (Elsevier, 2010). doi:10.1016/C2009-0-18081-9

24. Stoops, G. *Guidelines for analysis and description of soil and regolith thin sections*. (Soil Science Society of America, Inc. Madison, USA, 2003).

25. Goldberg, P. *et al.* Bedding, hearths, and site maintenance in the Middle Stone Age of Sibudu Cave, KwaZulu-Natal, South Africa. *Archaeol. Anthropol. Sci.* **1**, 95–122 (2009).

26. Bhattacharya, S., Dutta, S. & Summons, R. E. A distinctive biomarker assemblage in an Infracambrian oil and source rock from western India: Molecular signatures of eukaryotic sterols and prokaryotic carotenoids. *Precambrian Res.* **290**, 101–112 (2017).

27. Jambrina-Enríquez, M., Herrera-Herrera, A. V. & Mallol, C. Wax lipids in fresh and charred anatomical parts of the Celtis australis tree: Insights on paleofire interpretation. *Org. Geochem.* **122**, 147–160 (2018).

28. Brittingham, A. *et al.* Geochemical Evidence for the Control of Fire by Middle Palaeolithic Hominins. *Sci. Rep.* **9**, 15368 (2019).

29. Eglinton, G. & Hamilton, R. J. Leaf epicuticular waxes. *Science.* **156**, 1322–1335 (1967).

30. Rieley, G. *et al.* Sources of sedimentary lipids deduced from stable carbon-isotope analyses of individual compounds. *Nature* **352**, 425–427 (1991).

31. Otto, A. & Simpson, M. J. Degradation and preservation of vascular plant-derived biomarkers in grassland and forest soils from Western Canada.

Biogeochemistry **74**, 377–409 (2005).

32. Daura, J. *et al.* A 400,000-year-old Acheulean assemblage associated with the Aroeira-3 human cranium (Gruta da Aroeira, Almonda karst system, Portugal). *Comptes Rendus Palevol* **17**, 594–615 (2018).

33. Evans, M. E. . & Heller, F. *Environmental Magnetism: Principles and Applications of Environmagnetics*. (Academic Press, 2003).

34. Maki, D., Homburg, J. A. & Brosowske, S. D. Thermally activated mineralogical transformations in archaeological hearths: Inversion from maghemite γFe2O4 phase to haematite αFe2O4 form. *Archaeol. Prospect.* **13**, 207-227 (2006). doi:10.1002/arp.277
